# Inhomogeneities in 3D Collagen Matrices Impact Matrix Mechanics and Cancer Cell Migration

**DOI:** 10.3389/fcell.2020.593879

**Published:** 2020-11-05

**Authors:** Alexander Hayn, Tony Fischer, Claudia Tanja Mierke

**Affiliations:** Biological Physics Division, Faculty of Physics and Earth Sciences, Peter Debye Institute for Soft Matter Physics, University of Leipzig, Leipzig, Germany

**Keywords:** structural homogeneity, cancer, invasion, extracellular matrix, motility, atomic force microscope, elasticity, stiffness

## Abstract

Cell motility under physiological and pathological conditions including malignant progression of cancer and subsequent metastasis are founded on environmental confinements. During the last two decades, three-dimensional cell migration has been studied mostly by utilizing biomimetic extracellular matrix models. In the majority of these studies, the *in vitro* collagen scaffolds are usually assumed to be homogenous, as they consist commonly of one specific type of collagen, such as collagen type I, isolated from one species. These collagen matrices should resemble *in vivo* extracellular matrix scaffolds physiologically, however, mechanical phenotype and functional reliability have been addressed poorly due to certain limitations based on the assumption of homogeneity. How local variations of extracellular matrix structure impact matrix mechanics and cell migration is largely unknown. Here, we hypothesize that local inhomogeneities alter cell movement due to alterations in matrix mechanics, as they frequently occur in *in vivo* tissue scaffolds and were even changed in diseased tissues. To analyze the effect of structural inhomogeneities on cell migration, we used a mixture of rat tail and bovine dermal collagen type I as well as pure rat and pure bovine collagens at four different concentrations to assess three-dimensional scaffold inhomogeneities. Collagen type I from rat self-assembled to elongated fibrils, whereas bovine collagen tended to build node-shaped inhomogeneous scaffolds. We have shown that the elastic modulus determined with atomic force microscopy in combination with pore size analysis using confocal laser scanning microscopy revealed distinct inhomogeneities within collagen matrices. We hypothesized that elastic modulus and pore size govern cancer cell invasion in three-dimensional collagen matrices. In fact, invasiveness of three breast cancer cell types is altered due to matrix-type and concentration indicating that these two factors are crucial for cellular invasiveness. Our findings revealed that local matrix scaffold inhomogeneity is another crucial parameter to explain differences in cell migration, which not solely depended on pore size and stiffness of the collagen matrices. With these three distinct biophysical parameters, characterizing structure and mechanics of the studied collagen matrices, we were able to explain differences in the invasion behavior of the studied cancer cell lines in dependence of the used collagen model.

## Introduction

Metastasis caused by spreading of malignant cells represents the most harmful and dangerous aspect of cancer. Moreover, metastasis represents a prominent hallmark of the metastatic cascade (Pantel and Brakenhoff, 2004), that leads to secondary tumors at distant sites, which includes the capacity of cancer cells to efficiently invade different surrounding tissues (Gupta and Massagué, 2006), formed mainly by the specific extracellular matrices (ECMs). Changing the physical properties of the ECM protein meshwork (Krause et al., 2019) structures requires a highly adaptive behavior of invading cancer cells (Mierke, 2019a). Three-dimensional (3D) confined networks are relevant *in vitro* model systems to study cancer cell migration (Holle et al., 2019). Thereby adjustability and reproducibility represent a tunable and controlled microenvironment that is highly constructive to mimic ECM characteristics (Bersini et al., 2014) that cancer cells face *in vivo*.

In many studies, collagens of different origins or various collagen matrix compositions are utilized for 3D invasion assays. Implications for the migration behavior of cancer cells are the consequence. Howbeit, migration studies concerning cancer cell invasion into crafted 3D microenvironments are done in enormity, using several techniques and different materials (Hakkinen et al., 2011; Paul et al., 2016, 2017). In a growing number of studies, the spreading of cancer cells in defined environments is more focused on structural parameters and physical characteristics and their direct influence on the migratory behavior (Paszek et al., 2005; Wolf et al., 2009; Pathak and Kumar, 2012; Stroka and Konstantopoulos, 2014; Clark and Vignjevic, 2015; Carey et al., 2016; Um et al., 2017). Amongst others, inhomogeneity (Friedl and Alexander, 2011), matrix mechanics (Fischer et al., 2017) and confinements (Wolf et al., 2013) can drastically affect the migration potential.

Obviously, distinct parameters of the microenvironmental scaffold can stimulate and support the migration of specific cancer cells, whereas other parameters seem to rather constrain and impair the invasiveness of specific cancer cells (Wolf et al., 2013; Charras and Sahai, 2014). However, if these parameters impact the migration of all cancer cell types, or even all types of cells, in a universal manner is still on debate (Mierke, 2019b).

Apart from a broad field of techniques encompassing trans-well migration assays or 3D invasion assays and many more, the material of the engineered ECM plays an even more crucial role concerning the cancer cell migration on top and into these distinct microenvironments (Wolf et al., 2009). Engineered matrices polymerized of collagen type I, which is the most abundant ECM protein in mammals, serve as a physiological *in vitro* model system (Paul et al., 2016).

Since hydrogels are used to investigate cancer cell behavior, collagen type I from bovine dermis and rat tail tendon are prominently employed for matrix engineering (Brown, 1982; Behrens et al., 1989; Liebersbach and Sanderson, 1994; Friedl et al., 1997; Wolf et al., 2009, 2013; Willis et al., 2013; Mohammadi et al., 2015; Sapudom et al., 2015, 2019; Krause et al., 2019). In many cases, even mixtures of rat and bovine collagen are used (Koch et al., 2012; Lang et al., 2015; Lautscham et al., 2015; Fischer et al., 2017, 2020; Kunschmann et al., 2019; Riedel et al., 2019; Sauer et al., 2019; Mierke et al., 2020). Although those collagen matrices are made of the same type of collagen (namely type I), they can assemble to a totally different network exhibiting different physical properties (Wolf et al., 2009; Paul et al., 2016).

To what extend collagens of different origin and composition directly influence the cancer cell invasive phenotype, due to the altered biomechanical and topological properties of the various ECM systems, is mostly unknown. Thus, in this study, we analyzed three different collagen compositions for 3D cancer cell invasion, each of them at four different collagen concentrations. We compared the invasion behavior into these matrices for three different human breast cancer cell lines, such as MDA-MB-231, ZR-75, and MCF-7. Furthermore, we analyzed the matrix mechanics concerning elasticity and pore size of crafted 3D microenvironments varying in structural inhomogeneity. In fact, we found that the cancer cell invasion varies due to structural differences of these matrices. In specific detail, it has turned out that inhomogeneities of the 3D microenvironment, most importantly on the cell level, crucially influence the invasive phenotype of cancer cells.

## Results

### Characterization of Cell Line Specific Invasion in Different 3D Models

In order to obtain precise and distinct data for the invasion of human breast cancer cell lines, we generated different types of collagen networks from distinct collagen compositions. Therefore, we used commonly employed collagen compositions from collagen type I, such as pure collagens from rat tail (R) and bovine skin (B) and a 1:2 mixture of both (RB) collagen sources.

**FIGURE 1:**
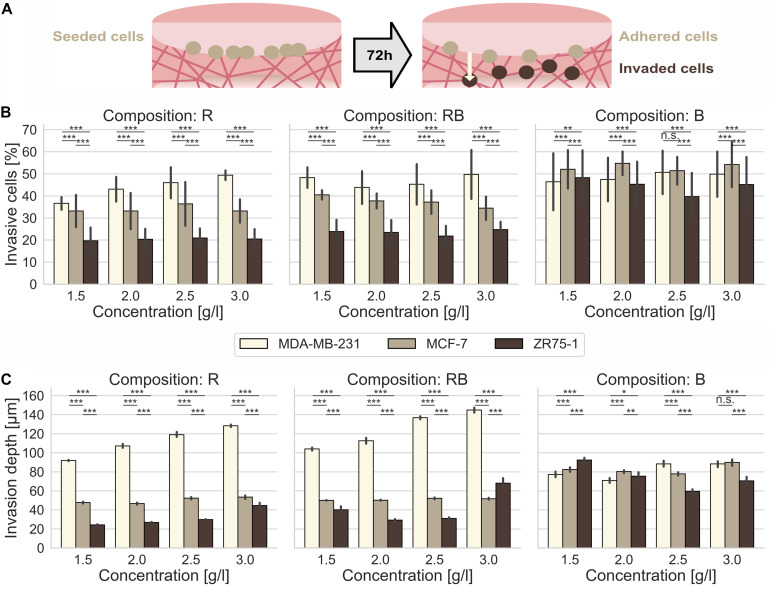
Cancer cell invasion. Effect of collagen monomer concentration on the invasiveness and invasion depth for human MDA-MB-231, MCF-7 and ZR75-1 breast cancer cells into crafted scaffolds of rat collagen (R), bovine skin collagen (B) and a 1:2 mixture or rat and bovine collagen (RB). **(A)** Sketch of the invasion assay. Cells are seeded on top of the collagen gels and invaded the matrices for 72 h. **(B)** The invasiveness and **(C)** the invasion depth for three observed human breast cancer cell lines in dependence of the collagen monomer concentration for three different collagen compositions (R, left; RB, middle; B, right). Data are presented as the means and SD for at least 5–12 repetitions in 4–6 independent experiments. Significance notions were derived from Welch’s unequal variance *t*-test, ***p ≤ 0.001, **p ≤ 0.01, *p ≤ 0.05, n.s., not significant. One-way ANOVA test revealed *** significance for all conditions **(A,B)**.

For a detailed insight in matrix dependent invasion, we altered the collagen concentrations from 1.5 g/l to 3.0 g/l, in steps of 0.5 g/l, respectively. By changing collagen concentration, we engineered loose (1.5 g/l), slightly loose (2.0 g/l), slightly dense (2.5 g/l) and dense (3.0 g/l) fibrillary networks. For all concentrations of all compositions, we seeded highly invasive MDA-MB-231, moderate invasive MCF-7 and minor invasive ZR75-1 cells separately on top of the collagen networks and let them invade for 3 days (Figure 1A). We analyzed the percentage of invasive cells (Figure 1B) and determined the invasion depths (Figure 1C) in dependence of the collagen composition and concentration. Further, we measured the invasion profiles (Supplementary Figures S1A–C), which show the probability for cells to be encountered in a certain depth also called their cumulative probability.

In summary, the cell line dependent invasion into R collagens exhibited a significantly higher invasive capacity (Supplementary Figure S1A) for MDA-MB-231 cells compared to a hindered invasion of MCF-7 cells and arrested ZR75-1 cells. The invasion of all three cell lines was somehow promoted at dense collagen matrices.

For R collagens, we found that MDA-MB-231 cells displayed a significantly (*p* = 0.001, or less) increased percentage of invasive cells (Figure 1B, left) with elevating collagen concentrations, from 36.6% ± 2.9% for loose collagen up to 49.4% ± 2.2% for dense collagens (Supplementary Table S1). In addition, the invasion depths (Figure 1C, left) increased significantly (*p* = 0.001, or less) with increasing concentration from 92.0 μm ± 7.1 μm for loose collagens up to 128.4 μm ± 11.9 μm for dense collagens. These results confirm the highly aggressive character of this cancer cell line. MCF-7 cells invaded R collagens with similar percentages of invasive cells at all concentrations, in the range of 33.1% ± 8.2% to 36.4% ± 9.9% (Supplementary Table S1). Invasion depths did not alter drastically in the range of 46.7 μm ± 8.8 μm up to 53.5 μm ± 14.5 μm (Supplementary Table S1). ZR75-1 cells invaded R collagens with stagnating percentages of invasive cells in the range of 19.7% ± 6.0% up to 21.0% ± 4.3% (Supplementary Table S1). Invasion depths were slightly but significantly (*p* = 0.001, or less) increased from 24.3 μm ± 6.1 μm in loose collagens up to 29.9 μm ± 5.3 μm in slightly dense collagens and considerably promoted in dense collagens (44.6 μm ± 29.7 μm). In summary, MDA-MB-231 cells invaded R collagens with significantly higher (*p* = 0.001, or less) percentages of invasive cells and penetrated these networks significantly deeper (*p* = 0.001, or less) at all concentrations compared to MCF-7 (1.5-fold invasion rate and 2.4-fold deeper invasion for dense collagens) and to ZR75-1 cells (2.4-fold invasion rate and 2.9-fold deeper invasion for dense collagen matrices).

MDA-MB-231 cells invaded with high percentages of invasive cells (Figure 1B, middle) into RB collagen matrices over all concentrations, in the range of 43.8% ± 7.4% up to 50.0% ± 11.1% invasive cells (Supplementary Table S1). Increasing collagen concentration significantly (*p* = 0.001, or less) promoted the invasion depths (Figure 1C, middle) from 104.1 μm ± 14.4 μm for loose collagens up to 145.0 μm ± 25.6 μm for dense collagens. However, for MCF-7 cells, we found a decreasing invasion ratio (Figure 1B, middle) with increasing collagen concentration, from 40.5% ± 2.1% for loose collagen matrices to 34.4% ± 5.3% for dense collagen matrices. Invasion depths (Figure 1C, middle) over all concentrations barely changed from 50.1 μm ± 2.8 μm up to 52.3 μm ± 8.7 μm (Supplementary Table S1). ZR75-1 cells invaded RB collagens less intense over all concentrations with invasion ratios (Figure 1B, middle) in the range of 23.5% ± 5.6% to 24.8% ± 3.6% (Supplementary Table S1). Invasion depths (Figure 1C, middle) were low for intermediate concentrations (2.0 g/l and 2.5 g/l), upshifted for loose and drastically increased for dense collagens (Supplementary Table S1). In summary, MDA-MB-231 cells aggressively invaded RB collagens deeply at high invasion rates. Compared to MCF-7 cells and ZR75-1 cells the percentage of invasive MDA-MB-231 cells and their invasion depths are significantly higher (*p* = 0.001, or less) at all concentrations. MDA-MB-231 cells invaded with a 1.2-fold (loose collagens) up to 1.5-fold (dense collagens) higher invasion rates compared to MCF-7 cells and a 2-fold (loose and dense collagens) higher invasion rate compared to ZR75-1 cells. The invasion depths of MDA-MB-231 cells were 2.1-fold (loose collagens) to 2.8-fold (dense collagens) higher compared to MCF-7 cells and 2.1-fold (dense collagens) up to 2.6-fold (loose collagens) higher than ZR75-1 cells.

The invasive potential (Supplementary Figure S1B) increased for MDA-MB-231 cells with increasing RB collagen concentration. For MCF-7 cells and ZR75-1 cells, no clear trend in the invasive potential could be seen. However, the invasion into RB collagen matrices was promoted at loose and dense collagen matrices by increased invasion rates and/or invasion depths compared to the intermediate concentrations for all three investigated cancer cell lines.

MDA-MB-231 cells invaded B collagens at a higher percentage of invasive cells (Figure 1B, right) over all concentrations, in the range from 46.4% ± 12.9% up to 50.7% ± 9.8%. In fact, their invasiveness slightly increased for denser collagen matrices (2.5 g/l and 3.0 g/l) compared to looser collagen matrices (1.5 g/l and 2.0 g/l). The invasion depths (Figure 1C, right) varied between 70.8 μm ± 25.1 μm and 88.4 μm ± 31.5 μm and were increased at denser collagen matrices (Supplementary Table S1). MCF-7 cells invaded these collagens (Figure 1B, right) with higher invasion rates (51.4% ± 6.3% up to 54.8% ± 5.4%) and deeper (Figure 1C, right) compared to MDA-MB-231 cells, with one exception at 2.5 g/l collagens. MCF-7 invasion depths are in the range from 77.9 μm ± 15.4 μm up to 90.0 μm ± 28.4 μm (Supplementary Table S1). ZR75-1 cells invaded B collagen matrices with high percentage of invasive cells (Figure 1B, right) and invasion depth (Figure 1C, right). Values decreased from 48.3% ± 12.4% and 92.4 μm ± 34.7 μm for loose collagen matrices to 39.7% ± 10.8% and 59.6 μm ± 25.8 μm for slightly dense collagens and again increased to 45.2% ± 12.5% and 70.6 μm ± 36.6 μm for dense collagen matrices. The invasiveness into B collagen matrices was promoted for each of the three cancer cell lines, which was underlined by their invasion profiles (Supplementary Figure S1C). However, MCF-7 cells invaded these collagen matrices with a higher invasion rate and predominantly deeper (except for 2.5 g/l collagens) compared to MDA-MB-231 cells. ZR75-1 cells displayed a promoted invasiveness at looser collagen matrices by means of invasion depth relating to the percent of invasive cells.

In fact, MDA-MB-231 cells invaded denser collagens more numerous and deeper for all observed collagen compositions (Supplementary Figures S2A,B). The ability of this cell line to penetrate 3D networks deeply was inhibited for networks solely made of bovine skin collagens, although their high invasiveness was not influenced by the collagen composition. MCF-7 cells invaded R and RB collagen matrices moderately in their invasiveness and invasion depth. Contrarily, they very aggressive hiked through B collagen matrices with high ratio of invasive cells and invasion depths (Supplementary Figures S2A,B). ZR75-1 cells were predominantly inhibited in the invasion of R and RB networks and considerable promoted in B collagen matrices due to their percentage of invaded cells and their invasion depths (Supplementary Figures S2A,B). Among all collagen compositions, dense collagen matrices promote these cells to invade surpassing deep.

### Effect of Collagen Composition on the Human Breast Cancer Collective Invasiveness

**FIGURE 2:**
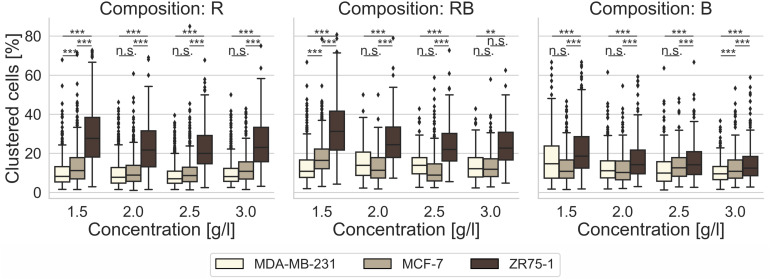
Cell clustering. Impact of cell line specific migration, as percentage of clustered cells among invaded cells, after 3 days of invasion into 3D collagen matrices of different monomer concentration and different collagen composition (R, left; RB, middle; B, right). Significance notions were derived from Welch’s unequal variance *t*-test, ***p ≤ 0.001, **p ≤ 0.01, n.s. not significant. Boxes are confined by 25th and 75th percentile, horizontal lines are the medians, whiskers describe 5th and 95th percentile. One-way ANOVA test revealed *** significance.

For the invasion into R collagen matrices (Figure 2, left) and RB collagen matrices (Figure 2, middle), we found no raised proclivity to migrate in collectives for MDA-MB-231 cells and for MCF-7 cells (Supplementary Table S1). On the opposite, ZR75-1 cells showed a significantly higher (*p* = 0.001, or less; *p* = 0.002 and *p* = 0.63 as exception for 3.0 g/l RB collagens) affinity to invade these collagen matrices at all concentrations collectively. For B collagen matrices (Figure 2, right) the affinity for MDA-MB-231 cells and MCF-7 cells to invade through collective migration forms was not altered contrary to the migration of ZR75-1. Obviously, for invading these networks the ratio of clustered cells among the invaded cells decreased with increasing concentration to the level of MDA-MB-231 cells and MCF-7 cells (Supplementary Table S1). Thus, the predominant number of invasive cells invaded B collagens as single cells for all observed cell lines.

Considering the cancer cell invasion as depicted above and concerning the differences and changes in migration mode for invading different 3D collagen microenvironments we focused on the characteristics of these networks assuming that there are underlying structural details that bias the differences in the observed migratory behavior.

### Collagen Composition Influences Structural Characteristics of Collagen Matrices

**FIGURE 3:**
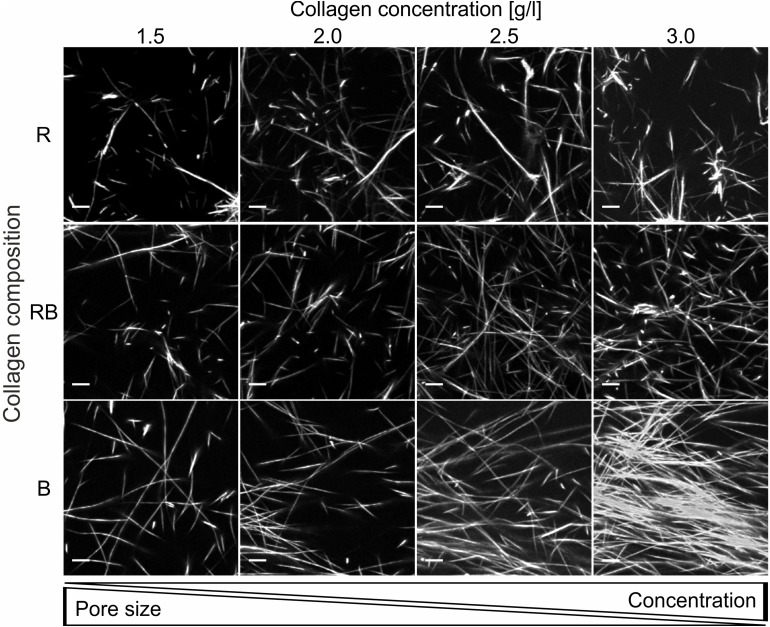
Collagen insight. Projection of representative collagen stacks for each concentration and composition. Dimensions are 50 μm × 50 μm and 10 slices (2.5 μm) are summed up. Scale bars are 5 μm. The collagen stacks were stained with TAMRA-SE for fluorescence and imaged with a CLSM using a 40x NA/1.10 water immersion objective.

Analyses of representative confocal laser scanning microscopy (CLSM) image stacks provided adequate insight into different structures (Figure 3) with increasing collagen monomer concentrations for R, RB, and B collagens. Obviously, R collagens formed distinct fibril bundles arranging progressively in dominantly developed node structures with increasing concentration. RB collagen fibrils arranged in apparent finer bundles that distributed more laminar compared to R collagens and at least formed large node-like structures. In contrast to R and RB networks, B collagen fibrils arranged in consecutive compact and dense node structures that dominated wide areas at an increasing rate with increasing concentration.

**FIGURE 4:**
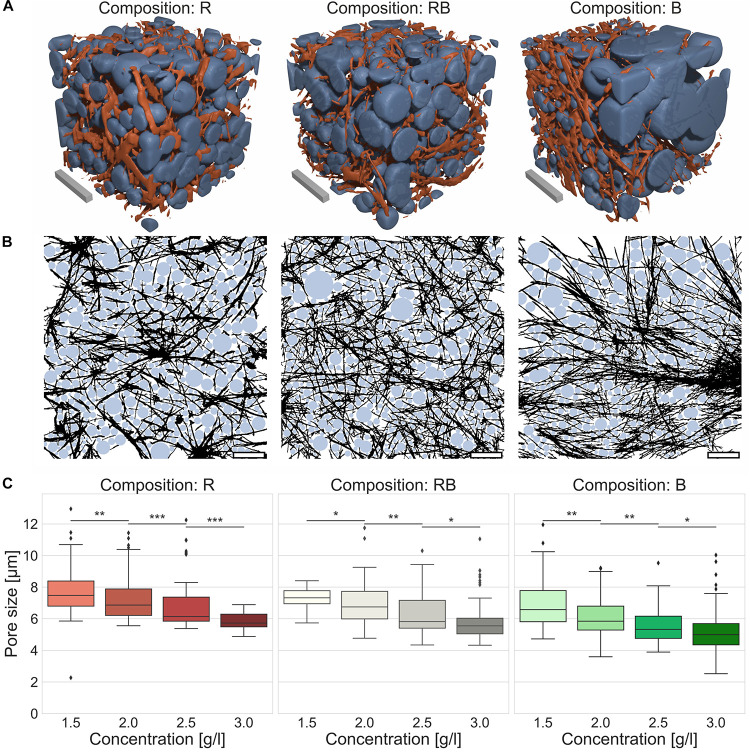
Pore size. Pore size as a crucial parameter to determine matrix properties. **(A)** Visualizations of detected pores of the collagen fiber matrices were assumed as spherical bubbles (blue) betted amongst collagen fibers (orange) for different collagen compositions (Rat (R), left; Rat/Bovine (RB), middle; Bovine (B), right). Scale bars are 20 μm. **(B)** Graphical realization (2D) of detected pores in exemplary image stacks for the three different collagen compositions (R, left; RB, middle; B, right). Scale bars are 20 μm. **(C)** Measurements of the pore size for the three collagen compositions conditional on the collagen monomer concentration. Significance notions were derived from Mann-Whitney *U* test, ***p ≤ 0.001, **p ≤ 0.01, *p ≤ 0.05, n.s., not significant. Boxes are confined by 25th and 75th percentile, horizontal lines are the medians, whiskers describe 5th and 95th percentile. Kruskal–Wallis test revealed *** significance.

For reliable insight into network characteristics, we analyzed the structure of R, RB and B collagens considering network pore size as a crucial parameter. Therefore, we recorded 3D cubic image stacks (Figure 4A) with a CLSM and analyzed them with an advanced pore size analysis (Figure 4B), published previously (Fischer et al., 2019). We found that all observed collagen compositions featured networks with significantly decreasing pore sizes by increasing collagen concentrations (Supplementary Table S2) (*p* = 0.01, or less for R collagens, *p* = 0.05 or less for RB and B collagens). The R collagen matrix pore size (Figure 4C) decreased from 7.7 μm ± 1.4 μm for loose collagen matrices to 5.8 μm ± 0.4 μm for dense collagen matrices. The pore size of RB collagen matrices (Figure 4C) decreased from 7.3 μm ± 0.7 μm for loose collagen matrices to 5.9 μm ± 1.3 μm for dense collagen matrices. B collagen pore size (Figure 4C) decreased from 6.9 μm ± 1.5 μm for loose collagen matrices to 5.2 μm ± 1.6 μm for dense collagen matrices.

**FIGURE 5:**
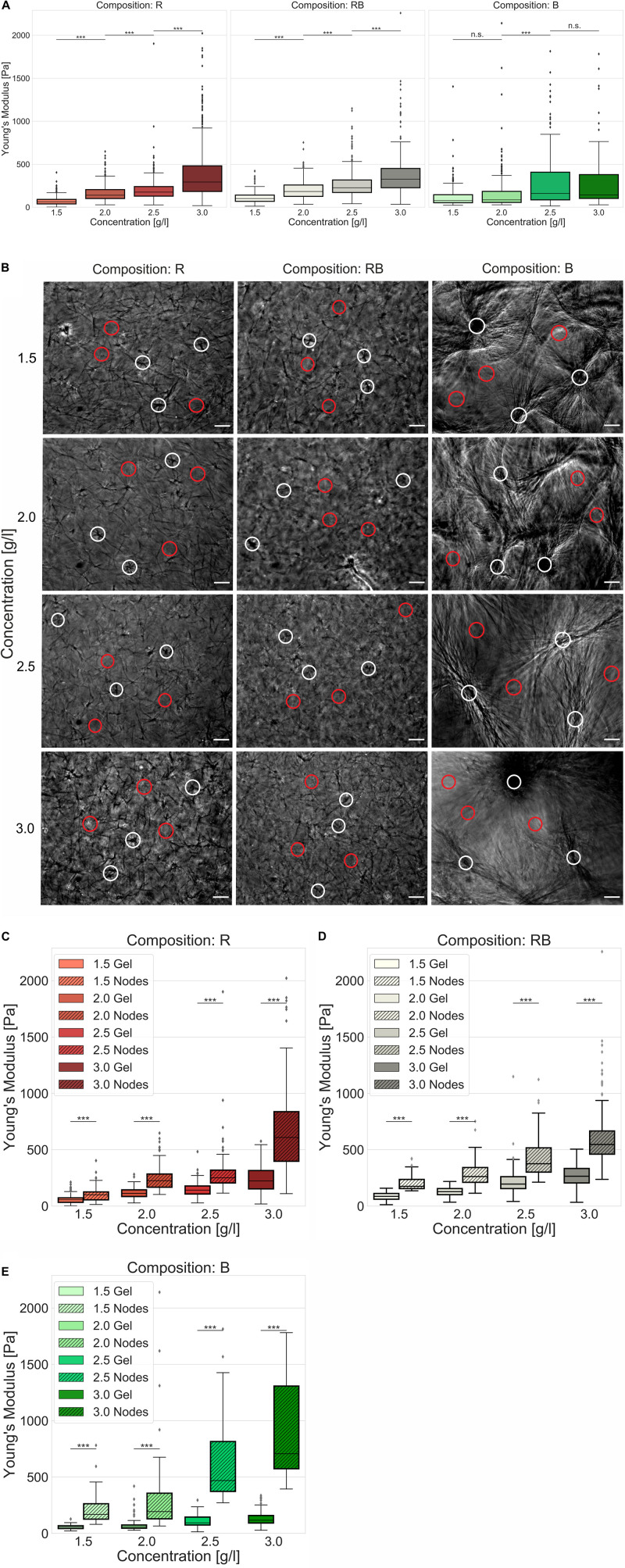
Elasticity. Elasticity of engineered collagen matrices. **(A)** Young’s modulus (stiffness) was ascertained by scanning force measurements (realized by a tipless cantilever attached with a 45 μm bead) for different collagen compositions (R, left; RB, middle; B, right) dependent on the collagen monomer concentration. **(B)** Exemplary phase contrast images of collagens that were used for AFM measurements. Representative indentations at node-containing regions of the collagen matrices were marked as white circles and indentations at gel regions of the collagen matrices were marked as red circles for different collagen compositions and collagen monomer concentrations. Scale bars are 50 μm. **(C–E)** Advanced stiffness consideration with focus on soft gel ranges and stiff node-like areas for **(C)** R collagen networks **(D)** RB collagen networks and **(E)** B collagen networks dependent on the collagen monomer concentration. Significance notions were derived from Mann–Whitney *U* test, ***p ≤ 0.001, n.s., not significant. Boxes are confined by 25th and 75th percentile, horizontal lines are the medians, whiskers describe 5th and 95th percentile. Kruskal–Wallis test revealed *** significance for all conditions **(A,C–E)**.

Another essential parameter for characterizing collagen networks is their elasticity in terms of stiffness (namely Young’s Modulus). Therefore, we used an atomic force microscopy (AFM) device and a well-accepted technique (Sapudom et al., 2015, 2019; Fischer et al., 2017). Thereby, we indented the collagen networks with a modified cantilever and measured their elastic response (Figure 5A). We found that R collagens significantly (*p* = 0.001, or less) increased their stiffness with increased collagen concentration from 63.0 Pa ± 48.5 Pa for loose collagen matrices up to 292.9 Pa ± 321.9 Pa for dense collagen matrices. Likewise, RB collagen matrices were significantly stiffer (*p* = 0.001, or less) due to increased concentration, namely 101.2 Pa ± 68.5 Pa for loose collagen matrices up to 326.2 Pa ± 260.1 Pa for dense collagen matrices. The stiffness of B collagens significantly (*p* = 0.001, or less) changed from looser to denser collagen matrices but showed no significant change between 1.5 g/l and 2.0 g/l (*p* = 0.09) as well as between 2.5 g/l and 3.0 g/l (*p* = 1.12) collagen matrices. The Young’s modulus amounted to 76.1 Pa ± 135.7 Pa (1.5 g/l), 84.6 Pa ± 256.3 Pa (2.0 g/l), 159.0 Pa ± 333.9 Pa (2.5 g/l) and 141.5 Pa ± 501.7 Pa (3.0 g/l). In summary, this contemplation characterized RB collagen matrices as being stiffer compared to R collagen matrices and B collagen matrices over the observed concentration spectra.

In fact, R and RB collagen matrices were predominantly stiffer than B collagen matrices (Supplementary Table S2) which is at the first glance conflictive to their visual appearance (Figure 5B) and the results of the pore size determination linking smaller pores to higher collagen concentrations (Supplementary Figure S3). Due to elasticity measurements, it is undisputed that higher concentrated collagen matrices are stiffer than lower concentrated collagen matrices and thus smaller pores can be linked to stiffer gels. Thus, one would expect the highest stiffness for B collagen matrices with the smallest pores (Supplementary Figure S3) among the observed collagen compositions. Analyzing elasticity measurements unpretentiously (assuming collagens being homogeneous) leads to such contrariety. The importance for analyzing structural characteristics reasonably was to estimate the necessary grade of accuracy or an adequate structure-centered view. In this case, we decided to analyze near the cell level by considering the inhomogeneities within the collagen matrices.

Applying an advanced approach, considering gel and node-like areas (Figure 5B), we observed significantly (*p* = 0.001, or less) different elasticities among all collagen concentrations and compositions (Figures 5C–E). Node-like areas in R collagen matrices (Figure 5C) were 1.8-fold to 2.7-fold stiffer than their corresponding softer matrix counterparts. The stiffness of R collagen matrices increased from 55.4 Pa ± 32.3 Pa for loose collagen matrices up to 222.7 Pa ± 118.5 Pa for dense collagen matrices. Node stiffness increased from 100.0 Pa ± 58.5 Pa for loose collagen matrices up to 608.9 Pa ± 390.4 Pa for dense collagen matrices (Supplementary Table S2). Gel stiffness increased for RB collagen matrices (Figure 5D) from 85.7 Pa ± 34.1 Pa for loose collagens up to 264.3 Pa ± 151.0 Pa for dense collagens, whereas the stiffness of node-like areas were 1.9-fold to 2.1-fold stiffer and increased from 174.5 Pa ± 72.1 Pa for loose collagen matrices to 546.5 Pa ± 285.1 Pa for dense collagen matrices. B collagen matrices (Figure 5E) possessed 3.0-fold to 6.0-fold stiffer node-like areas exhibiting a matrix stiffness increasing from 56.1 Pa ± 17.5 Pa for loose collagen matrices up to 116.9 Pa ± 77.7 Pa for dense collagen matrices. Node stiffness increased from 168.8 Pa ± 170.4 Pa for loose collagen matrices up to 706.4 Pa ± 667.4 Pa for dense collagen matrices.

In summary, RB collagen matrices were stiffer than R collagen matrices in mostly all cases and B collagen matrices possessed the highest ratio between stiffer and softer areas and at denser collagens the stiffest nodes and softest gels. Finally, the increasing fraction of B collagen concentration stiffened the networks at apparent spots (node-like areas). For B collagens, the collagen monomers relocated at compact and dense structures and soft gel counterparts. R collagens formed networks with largest pores and a less stiff structure. RB collagen matrices are found somehow in between R and B collagen matrices, when considering pore size and elasticity. These findings justify and necessitate the introduction of another biophysical parameter to distinguish the network homogeneity.

**FIGURE 6:**
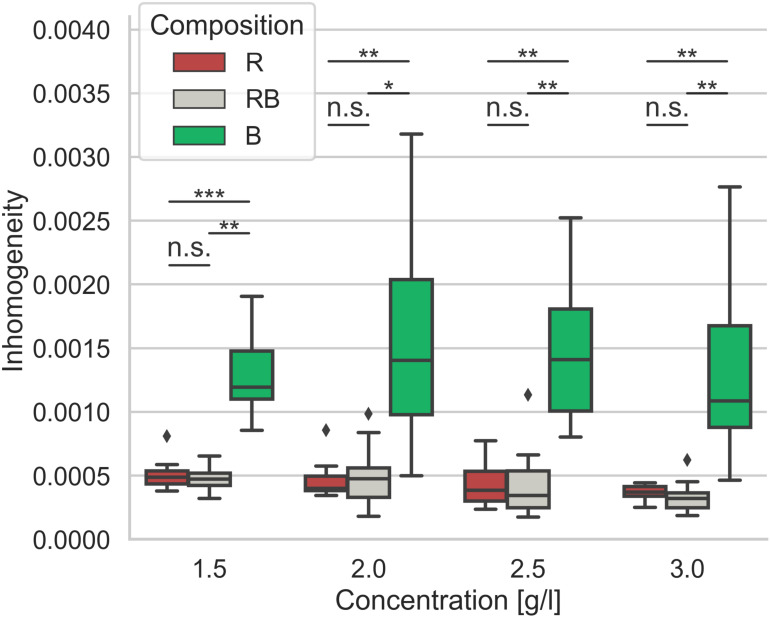
Inhomogeneity. Matrix scaffold inhomogeneity of networks of different collagen compositions for concentrations ranging from 1.5 g/l to 3.0 g/l. Each boxplot contains data of at least 8 – 13 image stacks obtained from different independent measurements. Significance notions were derived from Mann-Whitney *U* test, ***p ≤ 0.001, **p ≤ 0.01, *p ≤ 0.05, n.s., not significant. Boxes are confined by 25th and 75th percentile, horizontal lines are the medians, whiskers describe 5th and 95th percentile. Kruskal–Wallis test revealed *** significance.

Salient differences in the distribution of collagen structures arise in dependence of the collagen composition. To analyze these differences, we investigated the collagens regarding their matrix scaffold inhomogeneity and introduced a novel parameter representing the structural inhomogeneities of the different networks (Figure 6). Obviously, the networks solely made of rat collagen and the collagens mixed from rat and bovine collagen differ significantly (*p* = 0.05 or less at 2.0 g/l, else *p* = 0.01 or less) in their matrix scaffold inhomogeneity compared to collagens solely made of bovine collagens at all concentrations. Between rat and mixed collagens no significant (*p* = 0.72 and higher) difference could be measured. Likewise, there is no significance within the single collagen compositions over the concentration spectra (*p* = 1.55 and higher). Although a slightly trend to decreased matrix scaffold inhomogeneity with increased collagen concentration can be observed.

## Discussion

The invasion of cancer cells is a complex process. The change of the cell shape (Lyons et al., 2016; Baskaran et al., 2020), the softening the cell body and/or the nucleus (Fischer et al., 2020), the enzymatic-degradation of the matrix (Gialeli et al., 2011; Wolf and Friedl, 2011), the switch between migration modes (Friedl and Wolf, 2010) are some of well-founded methods that cancer cells use to overcome steric barriers and thus efficiently invade confined networks.

In the past, the majority of cancer cell migration and invasion assays have utilized homogeneous, such as flat two-dimensional substrates (Ridley et al., 2003; Geiger et al., 2009), rather than inhomogeneous 3D extracellular matrix scaffolds. In almost all 3D motility assays based on extracellular matrix, it was assumed that the microenvironment is homogeneous on the cellular scale. In our study, dealing with the mechanical characteristics of 3D collagen matrices, we observed a discrepancy within the literature that no single homogeneous mechanical parameter reflects the mechanical phenotype of the matrices.

Every reaction that cancer cells show is forced by the interaction with their environment. It is accepted that a certain stiffness is needed to exert adherence-based forces to the matrix components (Paszek et al., 2005; Wisdom et al., 2018). Furthermore, a tradeoff between pore size and stiffness is crucial for invasion (Lang et al., 2015). Therefore, it is useful to observe the invasion of cancer cells and gain insights into structural and mechanical properties of the microenvironment, as these support the invasion of the cancer cells. Cancer cell invasion into collagen model systems is an important method to characterize the spread of cancer. It is known that cancer cells successfully invade different tissues *in vivo* (Wolf and Friedl, 2011; Clark and Vignjevic, 2015). Collagen matrix models aim to mimic such different tissues *in vitro* (Wolf et al., 2009; Herrmann et al., 2014; Shamir and Ewald, 2014; Paul et al., 2016). It is accepted that alterations in matrix mechanics frequently occur in tissue scaffolds *in vivo* (Khadpekar et al., 2019) and inherently were altered in diseased tissues (Akhtar et al., 2011; Lu et al., 2012). To understand *in vivo* incidents with the help of *in vitro* models the inhomogeneity of the microenvironment is an important aspect.

For exhaustive investigations, we decided to examine the invasion of three human breast cancer cell lines, with different migratory potential, into three standard collagen models. Further, we observed the structure of the collagens to classify their mechanics and intrinsic topology to obtain an explanation for the determined invasion behavior.

The invasion of highly invasive MDA-MB-231 cancer cells into collagens from rat tail and collagens mixed from rat tail and bovine skin was as high as expected. Those networks could also mirror the moderate and inhibited invasion of intermediary invasive ZR75-1 and of weakly invasive MCF-7 cancer cells.

It was rather unexpected that the cell migration into bovine collagens of all three cancer cell lines was impaired. Highly invasive MDA-MB-231 (Supplementary Figure S6) cancer cells were hindered to penetrate the matrices deeply whilst weakly invasive MCF-7 (Supplementary Figure S7) cancer cells tend to invade bovine matrices excessively. Intermediary invasive ZR75-1 (Supplementary Figure S8) cells changed their migration from predominantly collective invasion to single cell migration accompanied by an aptness to invade bovine networks deeply with a high ratio of invasive cells.

In accordance with existing studies, we have shown that both stiffness and pore size govern the invasion of cancer cells into 3D-enclosed matrices (Wolf and Friedl, 2011; Lang et al., 2015; Sapudom et al., 2015; Fischer et al., 2017, 2020). These parameters are eligible to roughly describe the interplay of cancer cell invasion with the microenvironment on a global level. Due to the highly inhomogeneous structure of collagen gels, cancer cells that migrate in confined matrices directly and constantly face soft and stiff areas as well as large and small pores. The diversity of microenvironmental conditions from the perspective of the migrating cell could be seen as inhomogeneity on the cell level. Considering the inhomogeneities of 3D ECM model systems clarify structural dependencies for cancer cell migration.

Observing the stiffness of the collagen networks with conventional AFM approaches could not explain the variations as found in the invasiveness. Thereby a cantilever with an attached bead usually scans the surface of the collagen gels, regardless of the local structural differences present in these gels. In this way, no relevant differences in the elasticity of collagen networks with respect to their composition are found. In fact, this means an assumption of networks as homogeneous by over-simplifying structural conspicuousness. We hypothesized that cells with several μm expansion face inhomogeneities within the surrounding 3D microenvironment in form of less complex areas, such as pores or the fluid phase of the gels and high complex fibril arrangements or node-like structures. It is comprehensible, when indenting into less complex areas (distinguishing between apparent gel and node-like areas), that the elasticity is not the same as when indenting a high complex structure. Although we used simple assumptions of soft and stiff areas distributed at the surface of the collagen matrices, the significant differences confirmed and justified this approach. Subtle distinctions between stiffer and softer areas were constructive and revelatory. Thus, a more comprehensible analysis was feasible. Monitoring stiffer and softer areas, we found for all collagen compositions and all concentrations, significantly differ in their corresponding elasticity. In turn, such a focus depicts mechanical inhomogeneities. In fact, the preeminently inhomogeneous character especially of bovine networks was recognized and provided evidence for the changed invasion characteristics.

The pore size is another crucial parameter to determine structural traits (Fischer et al., 2019). Concerning confined scaffolds with spatial hindrances the pore size is a key value for describing a fibrillary network. Applying advanced 3D pore size analysis represents a solid method to characterize variations of different networks (Molteni et al., 2013; Münster and Fabry, 2013; Fischer et al., 2019; Sauer et al., 2019). The pore size specifies structural differences due to concentration variations within a certain collagen composition. Thus, it is conductive to find a measure for inherent structural factors. According to this, we found a decreased pore size with increasing collagen concentrations for all three collagen compositions. Trying to find an explanation for the different invasion characteristics based on the measured median pore size was not possible. Relying upon the median pore size would promote the expectation that aggressive cancer cells can penetrate all collagen compositions deeply. Furthermore, weakly invasive MCF-7 or intermediary invasive ZR75-1 cells should be hindered to invade bovine collagens in the same manner as found for their invasion into rat and mixed collagens. However, we found an oppositional behavior for all three cell lines, which obviously is caused by structural differences among the collagen compositions. Consequently, the pore size solitary is not able to indicate such structural dependencies. Nevertheless, the pore size is a statistically based parameter and thus blind for the inhomogeneity of the networks to what it is applied. This is especially remarkable for bovine collagen matrices, which have the smallest median pore size compared to the pure rat and rat-cattle mixture compositions. Factual, bovine networks possessed huge node-like entrapments containing a multitude of very small pores. On the contrary, in between the node-like areas very large pores are enclosed. Recapped, bovine collagens form exceedingly inhomogeneous networks. In such considerably inhomogeneous meshwork, the median pore size could not mirror structural details accurately.

The determined collagen networks within this study are all made from collagen type-I. Their differences in network formation, mechanics and matrix scaffold inhomogeneity evidently are connected to the origin of the different connective tissues they were extracted from. Collagen fibers from tendon are described as crimped and more uniform as the even fibers of the skin and thus exhibit different mechanical properties (Meyer, 2019) matched to the tissues they are part of. One major difference in the properties of reconstituted collagen I fibrillary networks from tendon or skin occurs due to the different extraction methods that are used to obtain collagen monomers (Demou et al., 2005; Wolf et al., 2009; Shinsato et al., 2020). More cross-linked skin collagens were additionally pepsinized to cleave telopeptides. This drastically influenced the self-assembly of fibrils to multimeric fibrils that further formed diffuse networks (Demou et al., 2005; Wolf et al., 2009). Early observations (Helseth and Veis, 1981) reported a telopeptide-dependent delayed speed compared with non-pepsinized collagens during the polymerization of telopeptide-poor fibrils which is in line with our turbidity observations (Supplementary Figure S5). The combination of telopeptide intact collagen with telopeptide lacking collagens drastically influenced the formation of the network (Helseth and Veis, 1981). The formation of our mixed collagens corresponds to that effect and further they form networks that are more similar to collagens solely made of rat tail collagen than to collagens solely made of bovine skin collagens on the cell level. This similarity between R and RB collagens is reflected in the invasive behavior of the investigated cell lines.

Understanding the influence of the microenvironment on the migration of cancer cells is essential. Here, we introduce a novel parameter that can reflect the matrix scaffold inhomogeneity of networks on the cell level. This parameter describes the inherent changes of the microenvironment among different collagen compositions. In line with this, the novel matrix scaffold inhomogeneity parameter correlates with the composition-related changes in the invasion profiles of all observed cell lines. Thus, this parameter serves as a reliable feature to describe the 3D microenvironment and valorizes elasticity and pore size characteristics.

It is obvious that more inhomogeneous networks force clustered cells to change their migration characteristics to a single cell dominated invasion. Other studies have shown that denser ECMs as well as decreased porosity leads to a change from single to collective cell migration (Haeger et al., 2014). Due to the matrix scaffold inhomogeneity found within bovine networks, which means local disturbance of the ECM density and thus locally increased porosity, we observed a similar effect for the ZR75-1 cells.

Moreover, the inhomogeneity of the microenvironment (mirrored by the novel matrix scaffold inhomogeneity parameter) seems to be crucial for the invasion depth that cancer cells achieved. This finding is in line with other studies concerning the ECM heterogeneity and ECM resistance (Talkenberger et al., 2017). Networks that are more homogeneous could be invaded significantly deeper by highly invasive MDA-MB-231 cancer cells, which predominantly prefer adhesion-based mesenchymal invasion. Mostly highly invasive cancer cells utilize strong adherence dependent mechanisms, which is observable e.g., by pronounced fiber displacements (Fischer et al., 2017), to squeeze through narrow confinements accompanied by several intracellular deforming mechanisms (Krause and Wolf, 2015; Fischer et al., 2020). We hypothesized that these mechanisms could be applied most efficiently to a homogeneous scaffold. Moreover, cells with amoeboid invasion preference were hindered to invade deeply and excessively into these homogeneous networks. For all three cell lines we found the respectively, opposite behavior for invasion into bovine collagen matrices. Consideration of the novel matrix scaffold inhomogeneity parameter directed to the hypothesis that the increased inhomogeneity of the networks, impairs the effects on invasion. In fact, the invasion depth of the highly invasive MDA-MB-231 cells was restricted, although their invasion rate seemed unaffected. However, the invasion of weakly invasive MCF-7 cells into inhomogeneous networks was promoted. As reported by others, the lack of focal adhesions enforces a switch to amoeboid-like migration forms (Friedl and Wolf, 2010; Liu et al., 2015). Local inhomogeneities are discontinuities in the structure of the networks. It can therefore be assumed that inhomogeneities are places where penetrating cells have no constant adherence option. Thus, a switch of their migration mode from mesenchymal to amoeboid, what means a mesenchymal-amoeboid transition (MAT) (Friedl and Wolf, 2003) appear reasonable. Using amoeboid-like migration as a path-finding (Wolf et al., 2003) migration mode to overcome inhomogeneities, such as collagenous barriers (Sabeh et al., 2004) in networks from pepsinized collagen can explain the almost equal invasion depths and rates of all three cell lines investigated within this study. It is reported as proteinase-independent form of migration (Friedl and Wolf, 2003; Wolf et al., 2003; Sabeh et al., 2004). In the case of MDA-MB-231 cells, this hypothesis is supported by detectable changes of the cell morphology (Supplementary Figures S6, S9, Supplementary Table S3, and Supplementary Videos S1–S9). In case of MCF-7 cells (Supplementary Figure S7) and ZR75-1 (Supplementary Figure S8) cells, the predominant roundish appearance of invaded cells was observed independent from collagen concentration or composition. Nevertheless, future studies might elucidate this matter and thus, explain the change of the invasion profiles for highly invasive MDA-MB-231 cells at bovine networks. For weakly invasive MCF-7 cells, the amoeboid-like invasion seems an advantage to percolate into networks that are more homogeneous. Thus, with increased inhomogeneity they can invade networks at higher rates more efficiently. Anyway, the invasiveness for all observed cell lines adjust to the microenvironment in consequence of changed matrix scaffold inhomogeneity.

Future studies should aim to find a certain shift in stiffness of node-like structures in combination with a correlating pore size that provokes the turnover of cancer cell invasion from weakly to highly invasive and from single to collective migration as well. A sophisticated characterization for stiff and soft network areas describing fibrillary arborization or alignments is promising. When identifying an inhomogeneity barrier that is able to screen inhibited or promoted invasion may provide a more reliable parameter to characterize and compare the migratory and invasive capacity of cancer cells in various ECMs encompassing pure rat, pure bovine and combined rat/bovine gels and may provide a pronounced impact in cancer cell research. Due to a high potential the observations of cancer cell response to an inhomogeneous microenvironment under influence of anti-metastatic and anti-invasion drugs (Gandalovièová et al., 2017) should be enhanced. Subsequent studies focusing on the inhomogeneity of human tissues *in vivo* and ECM models *in vitro* seem to be conducive and help to enlighten the complex metastatic cascade where cell migration and invasion is the central issue.

Finally, observing inhomogeneities on the cell level amplify structural dependencies that directly influence cancer cell invasion. Hence, the characterization of structural inhomogeneities is important to better understand cancer cell invasion. Defined inhomogeneous *in vitro* model systems seem to be more appropriate to mimic *in vivo* ECM and primary tumor microenvironments compared to the commonly employed homogenous ones. The insight to mechanical and structural inhomogeneities can reveal the interplay of cells with the microenvironment and possible explain an altered migration mode.

### Key Findings

• Human breast cancer cells MDA-MB-231, MCF-7 and ZR75-1 adjust their invasive capacities into different collagen matrices due to changing environmental conditions.
• Cancer cells are sensitive to local inhomogeneities.
• Mechanical inhomogeneities are present in 3D collagen gels and can be measured by AFM.
• Pore size and elasticity govern cancer cell invasion.
• Local inhomogeneities alter invasion into 3D collagen networks.
• A novel matrix scaffold inhomogeneity parameter serves to explain structural differences in 3D microenvironments on the cell level.

## Materials and Methods

### Cells and Cell Culture

Human breast cancer cell lines (MDA-MB-231, MCF7, and ZR-75-1) were purchased from ATCC-LGC-Promochem (Wesel, Germany). These cell lines were cultured under normal conditions in an incubator (37°C, 5% CO_2_, 95% humidity) using 4.5 g/l DMEM culture medium with additional 10% Calf Serum and 1% penicillin streptomycin (Biochrom, Berlin, Germany). At 75–80% confluency cells were harvested.

### 3D Collagen Matrices

Three types of extracellular matrix models were crafted for this study. Collagens solely comprised of rat tail monomers (4 g/l rat collagen type I, SERVA, Heidelberg, Germany), collagens containing only bovine skin monomers (4 g/l bovine collagen type I, Biochrom, Berlin, Germany) and collagens composed of a mixture of both collagen monomer types (rat tail and bovine skin) in a mass fraction of 1:2 were used for all collagen related experiments (Fischer et al., 2017, 2019, 2020; Kunschmann et al., 2019). For the polymerization of the monomer solution, a 1 M phosphate buffered solution containing disodium hydrogen phosphate (Sigma Aldrich, Cat. No. 71636), sodium dihydrogen phosphate (Sigma Aldrich, Cat. No. 71507) and ultrapure water were mixed keeping stable conditions (pH value 7.4, ionic strength 0.7, final phosphate concentration 200 mM). The components were kept at 0°C for mixing and finally added to 6-well plates for invasion assays, Petri dishes for elasticity measurements, 96-well plates for investigating the polymerization dynamics, or ibidi 24-well μ-plates for studying the pore size characteristics. Due to differences in the polymerization times (Supplementary Figure S5) R and RB collagen matrices were incubated for 2 h whereas B collagens were incubated for 5 h under normal conditions.

### 3D Invasion Assays

To investigate cancer cell invasion into 3D collagen networks we used collagens prepared as described above. As previously described (Fischer et al., 2017, 2020; Mierke et al., 2017; Kunschmann et al., 2019) we used 1.2 ml solution containing collagen and buffer for each well of a 6-well plate. Under normal conditions (95% humidity and 37°C) the collagens polymerized and form around 500 μm thick matrices. Consequently, the gels were treated three times by a rinsing procedure using Dulbecco’s phosphate buffered saline (PBS). Afterward 2 ml DMEM were added at each well and incubated over night at normal conditions. We added 50.000 cells per well on top of the collagens, harvested at confluence between 75 and 85% under treatment with 0.125% Trypsin/EDTA solution. The cells invaded the collagen matrices for 72 h (Figure 1A). Subsequently, we fixed the assay with 2.5% glutaraldehyde and stained the cell nuclei using 4 μg/ml HOECHST 33342 overnight. Fluorescent image stacks were recorded by a CCD camera (Orca-R2, Hamamatsu-Photonics, Munich, Germany) mounted with a 0.55x c-mount adapter, on an inverted microscope (DMI8000B, Leica, Wetzlar, Germany). A 20x objective and an A4 filter cube (Leica) were used. For each well, we obtained at least 100 image stacks received from a randomly selected 10 × 10 position grid. Focal plane distance (*z*-distance) was 4 μm. We analyzed the image stacks with a method already published (Fischer et al., 2017, 2020). Hereby we used a custom build python application based on elaborated algorithms for 3D image analysis and filtering. We defined cells on the surface of the collagen matrices and in the first two focal planes as non-invasive, in order to counterbalance minimal surface deviations. Cells found 12 μm below the surface and deeper are assumed invasive. Only stacks containing at least 25 cells were evaluated.

### Cluster Analysis

Image stacks that served for investigations of the cancer cell invasion were secondary analyzed concerning clustering. Therefore we used a method previously described (Kunschmann et al., 2019). In detail, we used the SciPy (Jones et al., 2001) DBSCAN algorithm that serves for identifying clusters as well as single cells (Ester et al., 1996; Schubert et al., 2017). Detected as a cluster are amassments of at least five cells within a 20 μm nuclei distance.

### Analysis of the Pore-Size Characteristics

We analyzed the pore size of the collagen matrices as previously described (Fischer et al., 2020) based on a custom-built python program already published (Fischer et al., 2019). In more detail, polymerized collagen matrices were stained overnight with TAMRA-SE (Sigma-Aldrich, Cat. No.: 21955) and afterward rinsed and stored in PBS. We generated with a confocal laser scanning microscope (Leica TCS SP8, Mannheim, Germany) under usage of a 40x NA/1.10 water immersion objective 3D image stacks in 150 μm cubic dimensions.

### Analysis of the Matrix Elasticity

To determine the elasticity of the collagen matrices, we used an AFM method as previously described (Sapudom et al., 2015). To specify, a tip-less cantilever was modified with a 45 μm polystyrene bead. Polymerized collagen matrices were indented using a maximum indentation force of 5 nN (Sapudom et al., 2015). For indentations, we randomly picked areas assumed as node-like or gel areas (Figure 5B). For each measurement, we observed a minimum of three different regions. Each region containing at least 10 node-like structures and 10 gel structures. The standard Hertz model was fitted to the retract part of the force distant curves.

### Inhomogeneity Analysis

Determining the matrix scaffold inhomogeneity of the examined collagen matrices is crucial to understand the differences in structure, mechanics and cell behavior in these hydrogels. In this study, a novel approach to determine the matrix scaffold inhomogeneity of collagen gels was developed. First, each recorded 3D image stack was divided into smaller parts of 30 μm, which has been considered to be roughly the size of the cell microenvironment. Each part, as well as the whole image cube, were analyzed regarding pore-size, number of pores and collagen volume as shown previously (Fischer et al., 2019), resulting in three key parameters for each part and the whole image cube. The determined parameters of each part were put in relation to the whole image cube as a percentage. Subsequently, the standard deviation of each parameter was calculated, and the Euclidean norm was determined. The resulting number is defined as the matrix scaffold inhomogeneity and is a measure of how much the local structure in the studied collagen matrices varies on the cell level.

### Statistical Analysis

All measurements were performed at least in triplicates if not stated otherwise. Statistical analyses were determined by one-way ANOVA and Welch’s unequal variance *t*-test analysis. For data not normal distributed, the Kruskal–Wallis and Mann-Whitney *U* test were applied.

## Data Availability Statement

The raw data supporting the conclusions of this article will be made available by the authors, without undue reservation.

## Author Contributions

AH performed the majority of the experiments and data analysis, and wrote the manuscript. TF performed the data analysis, wrote the custom-made programs, and interpreted the data. CM designed the experiments, analyzed and interpreted the data, and wrote the manuscript. All authors reviewed the manuscript.

## Conflict of Interest

The authors declare that the research was conducted in the absence of any commercial or financial relationships that could be construed as a potential conflict of interest.
