# Supplementary material for: Inhomogeneities in 3D Collagen Matrices Impact Matrix Mechanics and Cancer Cell Migration
Source: Front Cell Dev Biol. 2020 Nov 5;8:593879. doi: 10.3389/fcell.2020.593879 (PMC7674772; doi:10.3389/fcell.2020.593879)
Supplement: Supplementary file 1 [file Data_Sheet_1.PDF]

Supplementary Material: Inhomogeneities in 3D collagen matrices impact matrix mechanics and cancer cell migration

Alexander Hayn [1], Tony Fischer[1], Claudia Tanja Mierke [1*]

Table S1: Invasion and clustering values

Composition	Cell line	Concentration [g/l]	Invasive cells [%] mean	Invasive cells [%] ± SD	Invasion depth [µm] mean	Invasion depth [µm] ± SD	N	Clustered cells [%] median	Clustered cells [%] 25%	Clustered cells [%] 75%	n
R	MDA-MB-231	1.5	36.6	2.9	92.0	7.1	10	8.2	5.4	13.2	960
		2.0	43.0	5.6	107.2	18.2	8	7.8	4.8	12.8	981
		2.5	46.0	7.0	119.1	23.3	10	7.0	4.8	10.9	1548
		3.0	49.4	2.2	128.4	11.9	8	8.2	5.7	12.3	788
	MCF-7	1.5	33.2	7.3	47.8	8.3	8	11.2	6.9	17.9	855
		2.0	33.1	8.2	46.7	8.8	6	8.9	5.7	13.9	779
		2.5	36.4	9.9	52.4	11.4	8	8.7	5.6	12.9	944
		3.0	33.2	5.3	53.5	14.5	6	10.9	6.9	15.7	548
	ZR75-1	1.5	19.7	6.0	24.3	6.1	10	27.7	18.2	38.5	525
		2.0	20.4	4.8	26.9	5.3	8	21.7	13.2	31.6	481
		2.5	21.0	4.3	29.9	5.3	10	20.0	14.7	29.2	489
		3.0	20.5	4.5	44.6	29.7	8	23.1	15.8	33.3	410
RB	MDA-MB-231	1.5	48.3	4.7	104.1	14.4	9	10.9	7.7	16.7	708
		2.0	43.8	7.4	112.7	34.8	11	13.9	8.8	20.7	247
		2.5	45.3	9.2	136.9	19.3	13	13.9	9.6	17.9	370
		3.0	50.0	11.1	145.0	25.6	11	12.2	8.0	17.9	376
	MCF-7	1.5	40.5	2.1	50.1	2.8	5	16.4	12.2	22.2	655
		2.0	37.7	3.4	50.2	4.9	9	11.4	7.4	17.9	327
		2.5	37.2	5.3	52.3	8.7	9	8.9	5.9	14.7	462
		3.0	34.4	5.3	51.8	8.1	7	11.9	8.2	17.2	395
	ZR75-1	1.5	23.9	5.4	40.2	27.6	6	31.3	21.7	41.7	380
		2.0	23.5	5.6	29.3	8.8	8	24.5	17.9	33.5	180
		2.5	21.8	4.7	31.0	12.4	10	22.0	16.1	30.2	261
		3.0	24.8	3.6	68.2	48.2	8	22.7	16.7	30.8	162
B	MDA-MB-231	1.5	46.4	12.9	77.2	30.9	12	14.7	7.3	23.8	614
		2.0	47.5	9.9	70.8	25.1	10	11.1	7.5	16.2	361
		2.5	50.7	9.8	88.4	31.5	10	9.9	5.8	15.8	307
		3.0	49.9	10.3	88.3	27.9	10	9.6	6.6	13.3	301
	MCF-7	1.5	52.1	8.7	82.5	24.8	12	10.8	7.3	16.7	594
		2.0	54.8	5.4	80.3	14.9	8	10.3	6.5	16.1	370
		2.5	51.4	6.3	77.9	15.4	8	12.6	8.3	17.9	249
		3.0	54.3	10.3	90.0	28.4	8	10.8	7.4	16.7	330
	ZR75-1	1.5	48.3	12.4	92.4	34.7	12	18.6	12.5	28.6	1013
		2.0	45.3	10.3	75.5	36.5	10	14.3	9.6	21.7	543
		2.5	39.7	10.8	59.6	25.8	10	14.2	9.2	20.9	314
		3.0	45.2	12.5	70.6	36.6	10	12.5	8.6	18.4	433

Table S2: Pore size and mechanics

Composition	Concentration [g/l]	Pore size [µm] Median	25%	75%	n	N	Elasticity (Young's Modulus) [Pa] Median	25%	75%	n	Gel Elasticity (Young's Modulus) [Pa] Median	25%	75%	n	Nodes Elasticity (Young's Modulus) [Pa] Median	25%	75%	n	N
R	**1.5**	7.5	**6.8**	8.4	**104**	16	**63.0**	39.2	**91.4**	489	**55.4**	33.3	**72.9**	305	**100.0**	55.5	**126.1**	184	**8**
	**2.0**	6.9	**6.2**	7.9	**93**	16	**139.5**	101.5	**205.4**	452	**112.7**	85.3	**144.2**	283	**224.9**	167.8	**284.5**	169	**5**
	**2.5**	6.1	**5.8**	7.4	**70**	11	**175.6**	129.0	**240.2**	396	**141.3**	107.7	**178.8**	244	**250.3**	205.8	**319.3**	152	**5**
	**3.0**	5.7	**5.5**	6.3	**101**	13	**292.9**	183.8	**481.9**	550	**222.7**	152.3	**315.8**	351	**608.9**	398.2	**837.7**	199	**8**
RB	**1.5**	7.3	**6.9**	7.8	**41**	7	**101.2**	69.0	**141.1**	273	**85.7**	61.2	**112.1**	210	**174.5**	155.9	**236.9**	63	**4**
	**2.0**	6.7	**6.0**	7.7	**74**	12	**182.8**	126.2	**258.6**	232	**127.8**	101.8	**155.3**	117	**262.2**	211.1	**342.2**	115	**5**
	**2.5**	5.8	**5.4**	7.2	**55**	10	**226.6**	171.8	**316.9**	379	**195.1**	155.8	**260.4**	283	**375.2**	301.5	**517.2**	96	**6**
	**3.0**	5.5	**5.0**	6.0	**82**	12	**326.2**	227.1	**450.6**	404	**264.3**	205.8	**334.3**	281	**546.5**	460.9	**667.1**	123	**7**
B	**1.5**	6.6	**5.8**	7.8	**86**	8	**76.1**	52.6	**145.5**	230	**56.1**	44.1	**68.6**	137	**168.8**	127.3	**263.7**	93	**3**
	**2.0**	5.8	**5.3**	6.8	**66**	9	**84.6**	56.2	**184.3**	289	**59.8**	46.5	**75.1**	170	**193.3**	129.8	**356.9**	119	**5**
	**2.5**	5.3	**4.8**	6.2	**107**	11	**159.0**	85.5	**407.5**	181	**93.5**	75.1	**145.6**	110	**468.7**	372.9	**816.1**	71	**4**
	**3.0**	5.0	**4.3**	5.7	**58**	9	**141.5**	102.1	**379.4**	70	**116.9**	92.9	**158.9**	52	**706.4**	574.4	**1307.4**	18	**3**

Figure S1

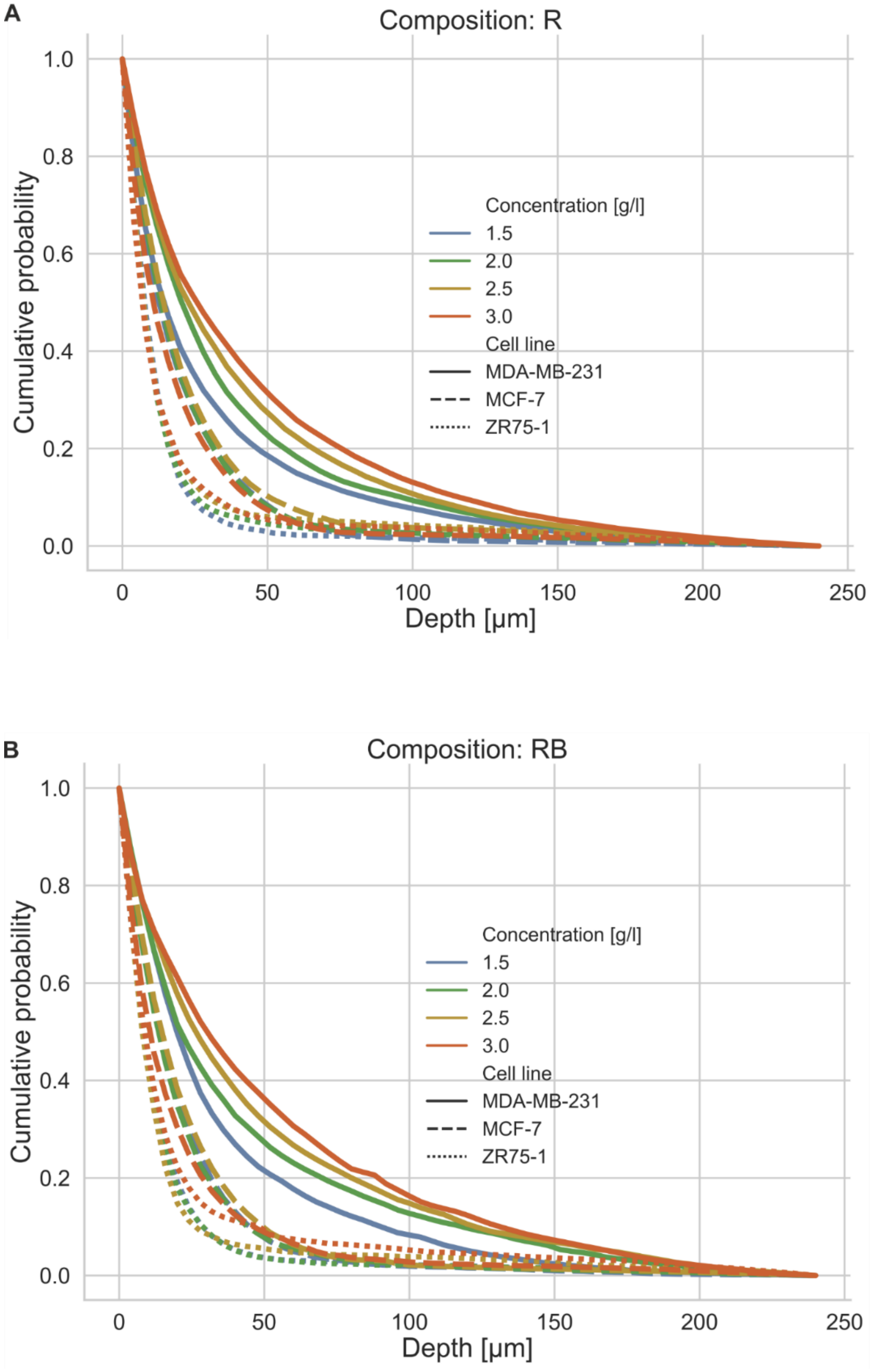
A
Composition: R
Cumulative probability
1.0
0.8
0.6
0.4
0.2
0.0
0
50
100
150
200
250
Depth [µm]
Concentration [g/l]
1.5
2.0
2.5
3.0
Cell line
MDA-MB-231
MCF-7
ZR75-1
B
Composition: RB
Cumulative probability
1.0
0.8
0.6
0.4
0.2
0.0
0
50
100
150
200
250
Depth [µm]
Concentration [g/l]
1.5
2.0
2.5
3.0
Cell line
MDA-MB-231
MCF-7
ZR75-1

C

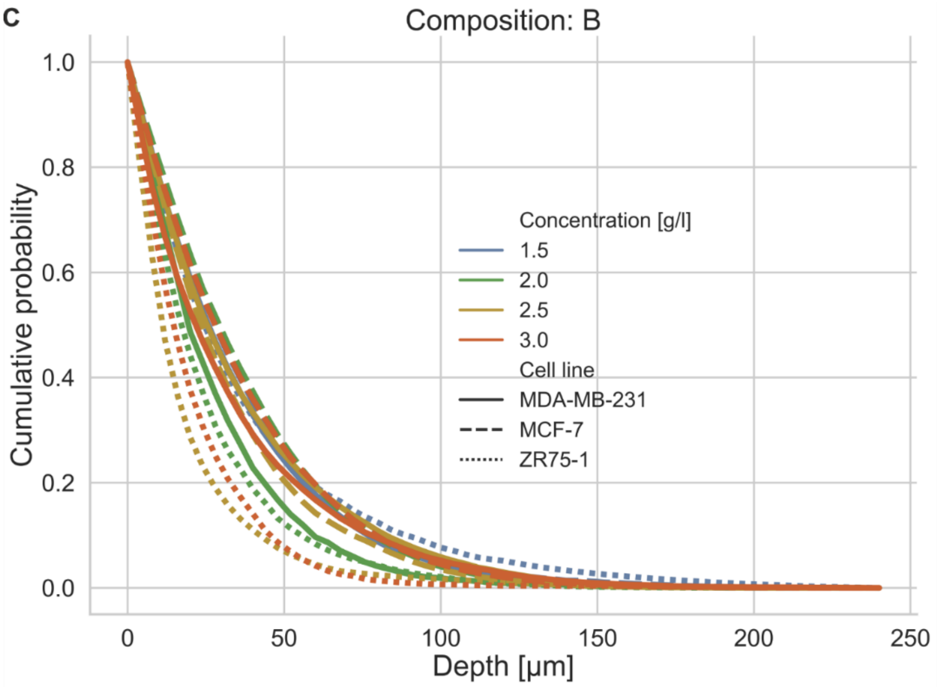

Figure S1: Invasion profiles for three human breast cancer cell lines. The cumulative probability shows the probability of cells found in a certain depth. Z-distribution of cells invaded **(A)** R collagens, **(B)** RB collagens and **(C)** B collagens.

Figure S2

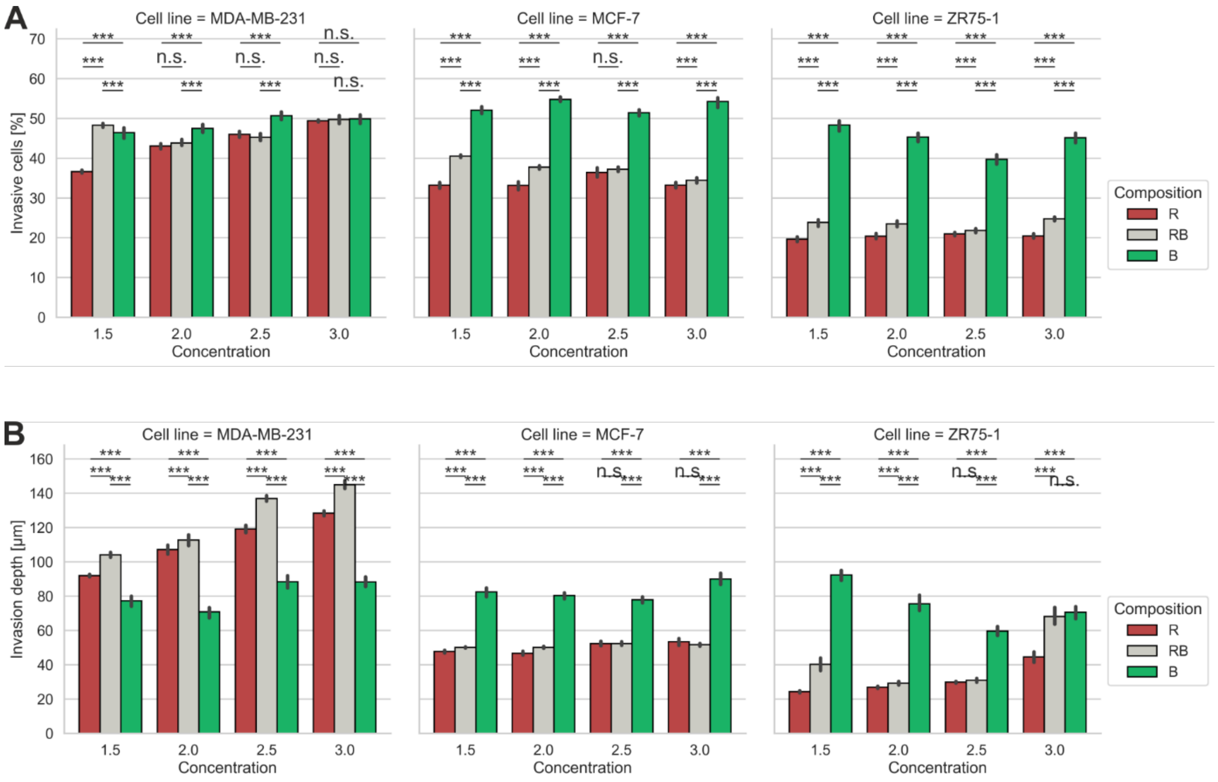

Figure S2: Invasiveness of three human breast cancer cells into different collagen networks with increasing collagen concentration. Different behaviors among the three collagen compositions are focused**. (A)** Ratio of invasive cells and **(B)** Invasion depth for MDA-MB-231 cells (left), MCF-7 cells (middle) and ZR75-1 cells (right). Significance notions were derived from Welch's unequal variance t-test, *** $p \leq 0.001$, n.s. not significant. One-way ANOVA test revealed *** significance for all conditions (Figure S2A-B).

Figure S3

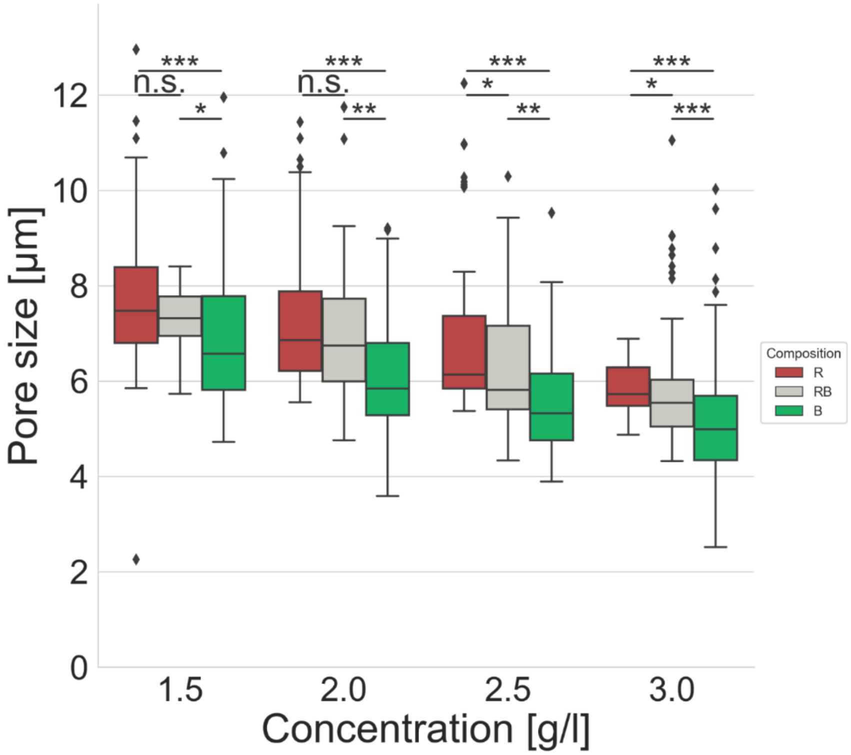

Figure S3: Pore size for three different collagen compositions with increasing collagen composition. In focus are the differences between the different compositions within a certain concentration. Significance notions were derived from Mann-Whitney U test, *** $p \leq 0.001$, ** $p \leq 0.01$, * $p \leq 0.05$, n.s. not significant. Boxes are confined by 25th and 75th percentile, horizontal lines are the medians, whiskers describe 5th and 95th percentile. Kruskal-Wallis test revealed *** significance.

Figure S4

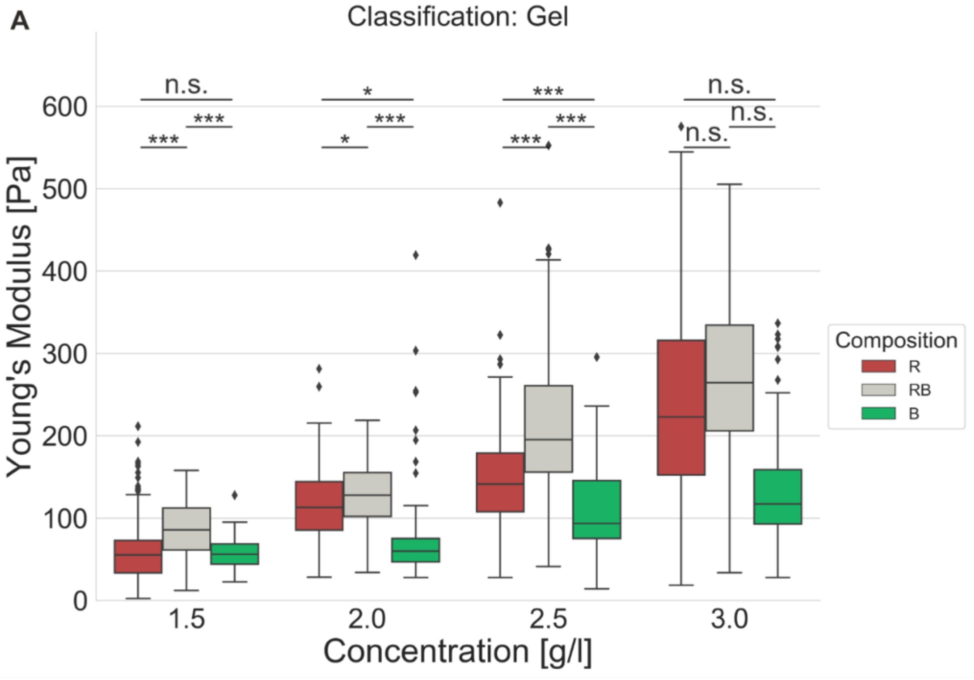

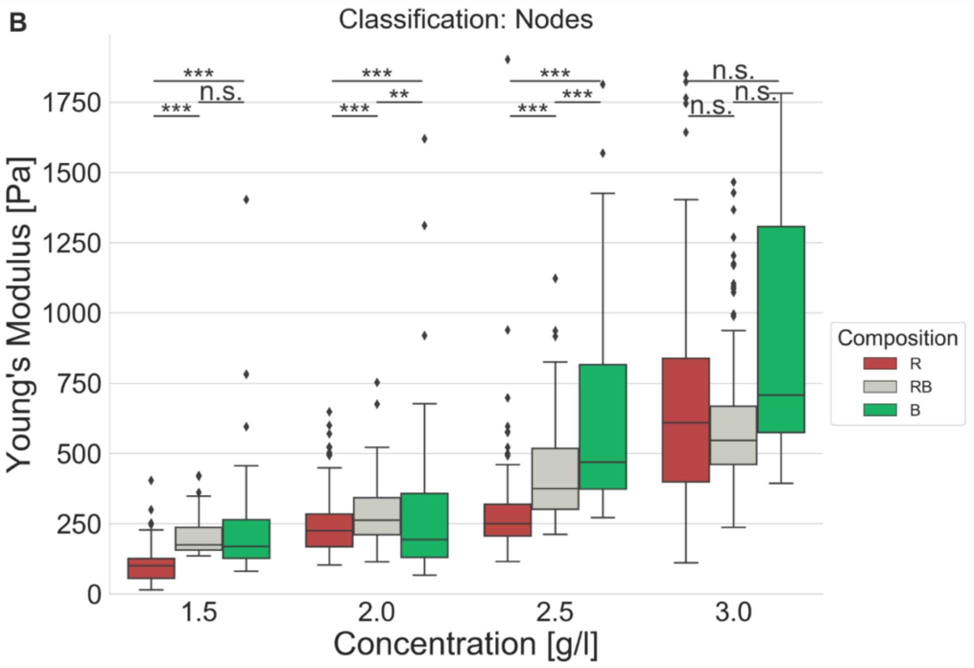

Figure S4: Elasticity of three different collagen networks with increasing collagen concentration. **(A)** Separate analysis of softer gel areas and **(B)** stiffer node-like areas show mechanical inhomogeneity. Data range limits of 0 to 600 Pa for gel areas and 0 to 2000 Pa for node-like areas are chosen for better visibility. Significance notions were derived from Mann-Whitney U test, *** $p \leq 0.001$, ** $p \leq 0.01$, n.s. not significant. Boxes are confined by 25th and 75th percentile, horizontal lines are the medians, whiskers describe 5th and 95th percentile. Kruskal-Wallis test revealed *** significance.

Figure S5

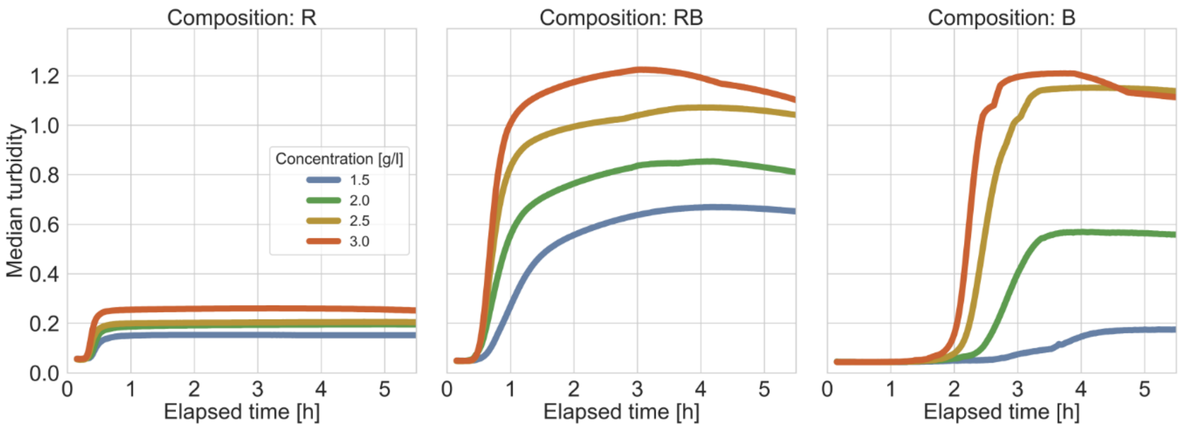

Figure S5: Polymerization dynamics for three different collagen networks. The turbidity measured with a plate reader device reveal differences in the fibrillation between R collagens (left), RB collagens (middle) and B collagens (right). Observing the network forming processes by polymerization, we used a microplate reader device and measured the turbidity. The fogginess is changed during the polymerization of the collagens. We found that R collagen networks polymerized faster and with increased turbidity for increasing collagen concentration. A short lag phase within the region of some minutes initiated a fast raising turbidity followed by a long steady state phase. However, RB collagen networks started measurably polymerization after a longer deferral (within 30-40 minutes) but higher turbidity among all concentrations compared to R collagens, but no clearly distinct plateau. B collagen networks need a long lag phase (1.5 hours to 2.5 hours) before fibrillation initiates. Loose (1.5 g/l) B collagens slowly polymerized with reaching a small maximum turbidity after 4.5 to 5 hours resulting in a plateau. Slightly loose (2.0 g/l) B collagens polymerized faster and with a higher turbidity compared to loose B collagens, higher than R collagens but lower than RB collagens. Slightly dense (2.5 g/l) and dense (3.0 g/l) B collagens polymerized progressively faster and with increased turbidity compared to looser collagens. Turbidity levels of denser B collagens are considerably higher than for R collagens and are at the level of RB collagen turbidity. Turbidity of each 100 µl collagen solution, prepared at 4°C in pre-cooled Greiner 96-well plates, were measured at 405 nm in intervals of 90 seconds over 5.5 hours at 37°C with a Synergy H1 plate reader device (BioTek, Winooski, VT, USA). Three independent measurements with each four repetitions were made.

Figure S6

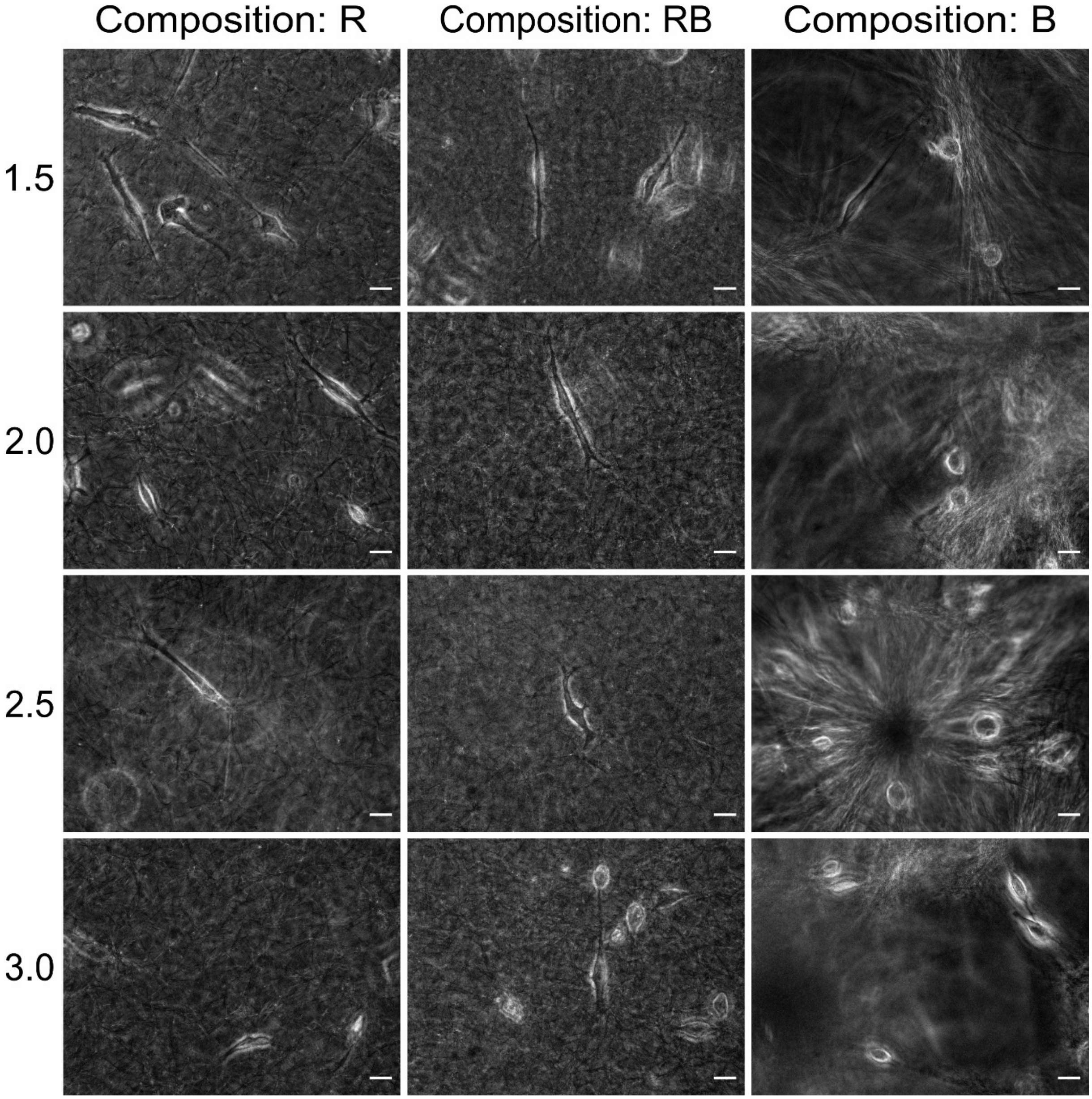

Figure S6: Phase contrast images of MDA-MB-231 cells after three-days invasion into different collagen matrices. Cells inside the collagen matrices composed of R collagen or the mixed RB collagen predominantly are found to be enlarged and spindle-shaped. Distinct protrusions are characteristic. Cells inside the collagen matrices of B collagens are found to be spindle-like and elongated, if adhered in between adjacent fiber bundles and node-like structures. In addition, an increased number of invaded cells appeared more roundish compared to cells invaded R and RB collagen matrices. Snapshots are recorded by a CCD camera (Orca-R2, Hamatsu-Photonics, Munich, Germany) mounted with a 0.55x c-mount adapter on an inverted microscope (DMI8000B, Leica, Wetzlar, Germany). A 40x objective was used. Scale bars are 20 µm.

Figure S7

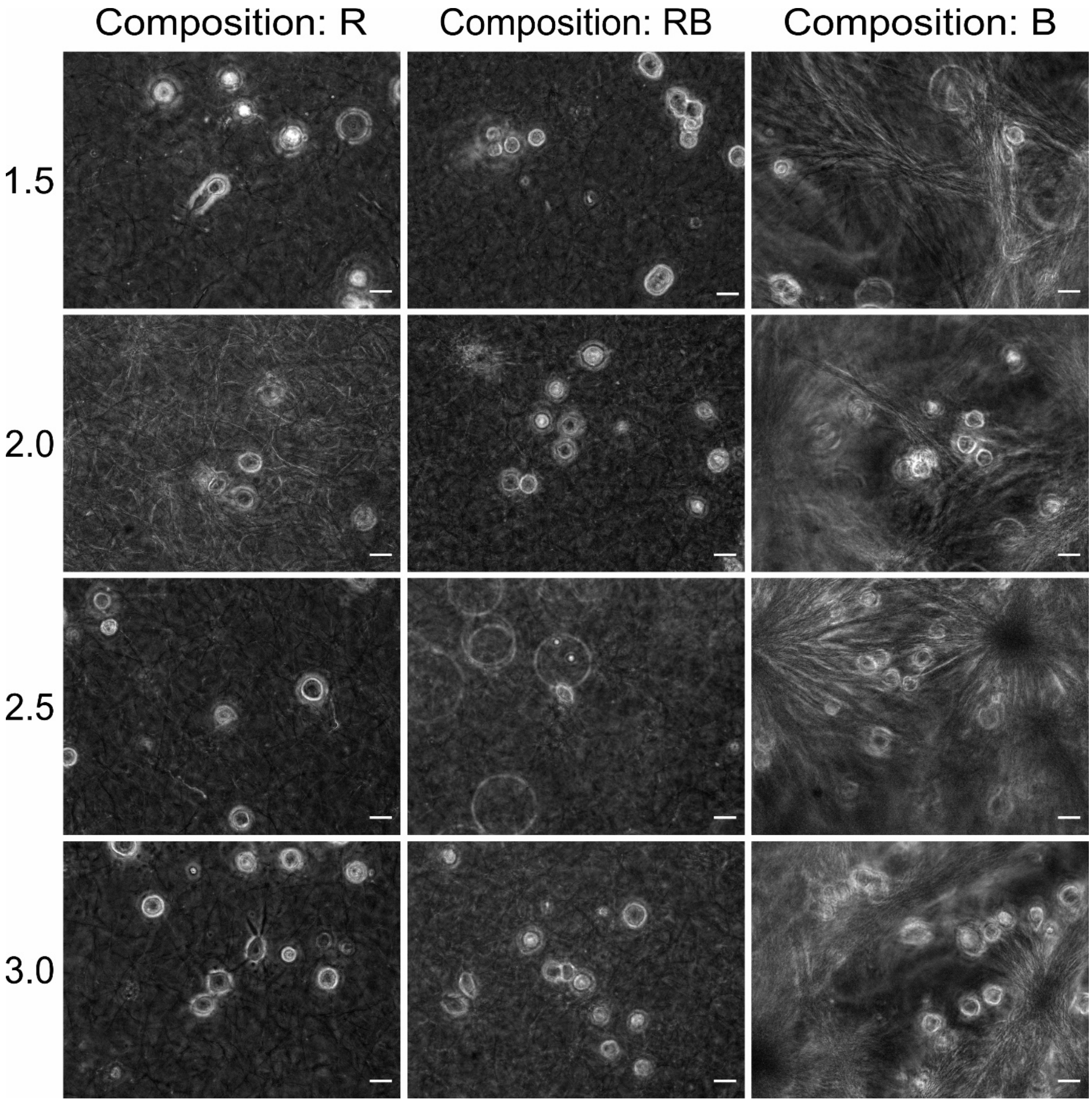

Figure S7: Phase contrast images of MCF-7 cells after three days-invasion into different collagen matrices. The typical round-shaped character of this cell line could be observed at all concentrations and all compositions. Snapshots are recorded by a CCD camera (Orca-R2, Hamatsu-Photonics, Munich, Germany) mounted with a 0.55x c-mount adapter on an inverted microscope (DMI8000B, Leica, Wetzlar, Germany). A 40x objective was used. Scale bars are 20 µm.

Figure S8

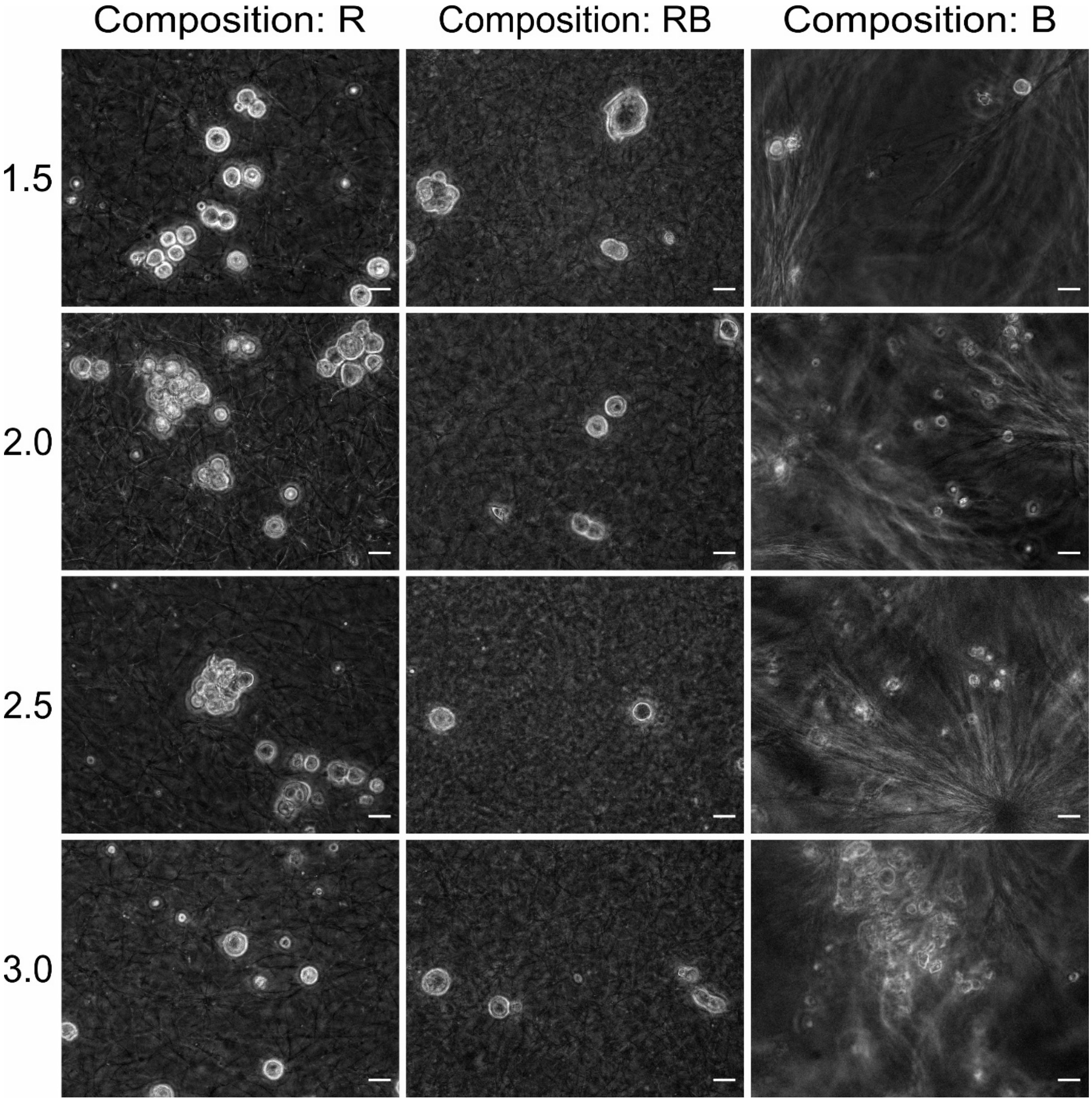

Figure S8: Phase contrast images of three-days collagen invasion studies with ZR75-1 cells. Different collagen matrices were used. Most cells shown here are at the surface of the collagens. Only a small amount of cells slightly invaded the matrices. The typical round-shaped character of this cell line could be observed at all concentrations and all compositions. Cells found at/ in R and RB collagens were often clustered. At B collagens more single cells are embedded into the inhomogeneous matrices compared to R and RB collagens. Snapshots are recorded by a CCD camera (Orca-R2, Hamatsu-Photonics, Munich, Germany) mounted with a 0.55x c-mount adapter on an inverted microscope (DMI8000B, Leica, Wetzlar, Germany). A 40x objective was used. Scale bars are 20 μm.

Figure S9

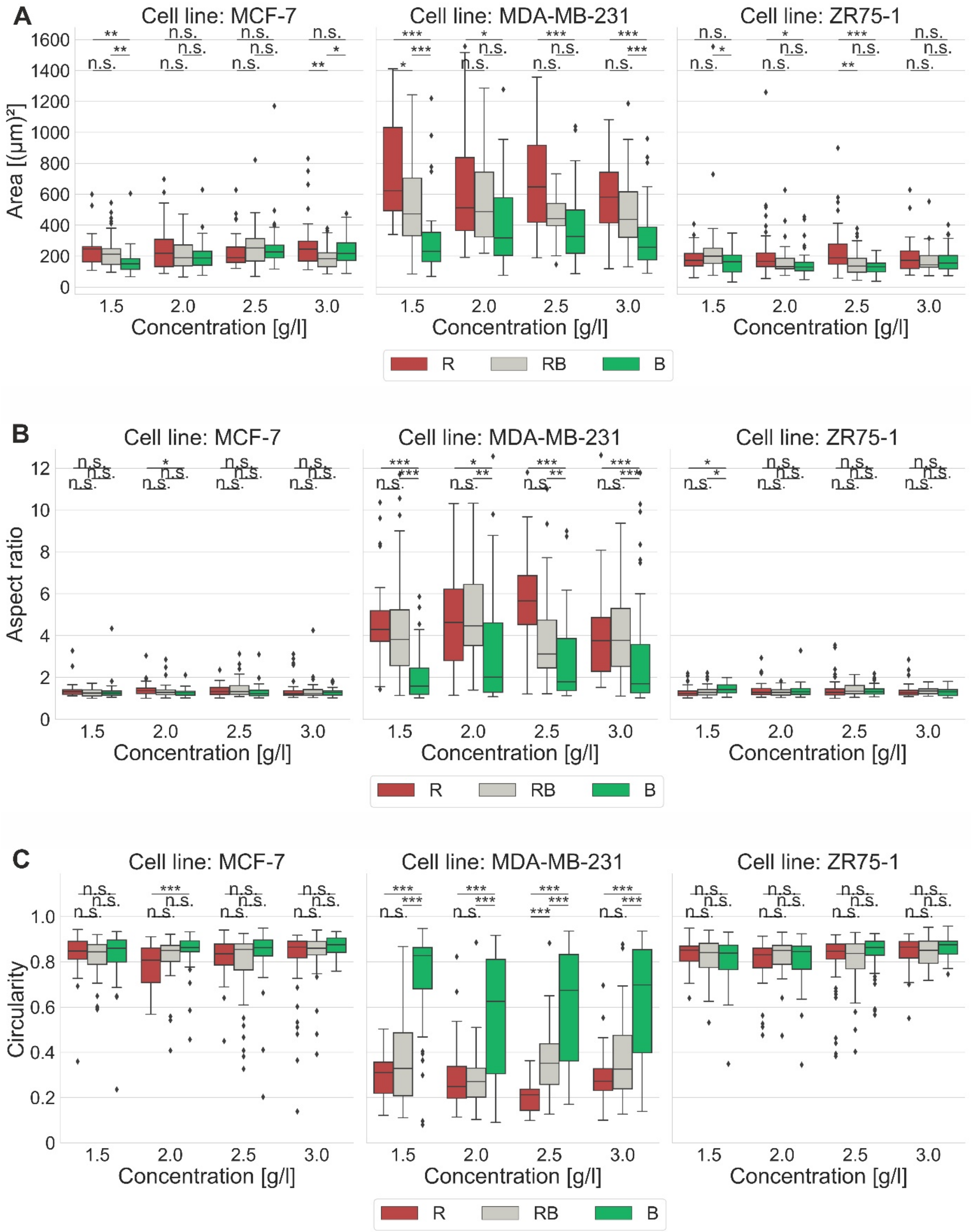

Figure S9: Cell morphology of invaded cells in dependence of collagen concentration and composition. For calculation of cell area **(A)**, aspect ratio **(B)** and circularity **(C)** for the three human

breast cancer cell lines at all conditions we analyzed at least 30 cells from three independent invasion measurements using ImageJ. Invaded MCF-7 cells, as well as ZR75-1 cells, show predominantly no significant differences in their area, aspect ratio or circularity over the concentration and composition spectra. On the contrary, MDA-MB-231 cells differ partially significant (especially in their aspect ratio and circularity) in their morphologic characteristics if compared R and B collagens, as well as RB and B collagens. The boxes determining the circularity of MDA-MB-231 cells in B collagens with collagen concentrations of 2.0 g/l and higher are widened due to the occurrence of spindle-like and roundish cells as well.

Table S3: Cell morphology

Composition	Cell line	Concentration [g/l]	count	Area [(µm)²] 25%	Area [(µm)²] 50%	Area [(µm)²] 75%	Circularity 25%	Circularity 50%	Circularity 75%	Aspect ratio 25%	Aspect ratio 50%	Aspect ratio 75%	apparent migration mode*
R	MDA-MB-231	1.5	30.0	493.06	621.91	1031.74	0.22	0.31	0.36	3.72	4.30	5.19	m
		2.0	30.0	365.12	512.95	836.91	0.20	0.25	0.34	2.81	4.63	6.22	m
		2.5	30.0	418.90	647.07	916.95	0.14	0.21	0.23	4.53	5.65	6.87	m
		3.0	46.0	415.36	581.74	742.18	0.23	0.27	0.33	2.29	3.76	4.86	m
	MCF-7	1.5	31.0	162.22	246.73	261.03	0.81	0.85	0.89	1.20	1.32	1.41	a
		2.0	40.0	131.53	219.07	308.66	0.71	0.81	0.86	1.23	1.37	1.49	a
		2.5	31.0	158.67	189.42	258.40	0.79	0.84	0.88	1.19	1.33	1.52	a
		3.0	49.0	168.57	244.97	296.18	0.82	0.87	0.89	1.16	1.22	1.37	a
	ZR75-1	1.5	35.0	136.76	173.08	216.49	0.80	0.85	0.87	1.14	1.24	1.36	a
		2.0	48.0	131.84	166.18	220.09	0.77	0.83	0.86	1.18	1.29	1.48	a
		2.5	63.0	147.26	188.26	278.48	0.81	0.85	0.88	1.12	1.29	1.46	a
		3.0	45.0	119.51	173.11	232.70	0.82	0.87	0.89	1.17	1.26	1.39	a
RB	MDA-MB-231	1.5	64.0	332.37	473.07	704.29	0.21	0.33	0.49	2.57	3.81	5.23	m
		2.0	31.0	324.35	487.38	743.09	0.20	0.27	0.33	3.52	4.47	6.45	m
		2.5	32.0	396.90	442.26	540.64	0.26	0.35	0.44	2.46	3.12	4.74	m
		3.0	40.0	321.47	437.34	615.49	0.24	0.33	0.48	2.53	3.78	5.29	m
	MCF-7	1.5	66.0	146.65	212.04	248.10	0.79	0.84	0.88	1.12	1.25	1.41	a
		2.0	41.0	137.57	189.31	272.91	0.80	0.85	0.87	1.18	1.29	1.41	a
		2.5	51.0	168.21	252.62	314.20	0.77	0.86	0.88	1.20	1.33	1.61	a
		3.0	56.0	131.28	183.13	220.77	0.83	0.86	0.89	1.16	1.24	1.42	a
	ZR75-1	1.5	43.0	153.00	199.25	251.47	0.78	0.84	0.88	1.17	1.28	1.43	a
		2.0	30.0	118.50	132.09	185.46	0.79	0.85	0.87	1.16	1.29	1.42	a
		2.5	37.0	96.18	135.54	185.46	0.77	0.84	0.88	1.23	1.34	1.62	a
		3.0	32.0	128.49	143.56	202.55	0.79	0.85	0.89	1.23	1.35	1.45	a
B	MDA-MB-231	1.5	43.0	165.06	230.32	353.79	0.68	0.83	0.86	1.20	1.58	2.45	a/m
		2.0	46.0	203.77	317.95	577.31	0.31	0.63	0.81	1.30	2.01	4.61	a/m
		2.5	55.0	218.02	327.69	497.67	0.36	0.68	0.83	1.37	1.80	3.87	a/m
		3.0	59.0	175.92	257.49	387.39	0.40	0.70	0.85	1.27	1.70	3.58	a/m
	MCF-7	1.5	43.0	115.04	149.95	183.12	0.80	0.86	0.90	1.15	1.25	1.36	a
		2.0	65.0	141.19	187.81	230.90	0.85	0.86	0.89	1.15	1.27	1.34	a
		2.5	58.0	188.83	226.59	271.05	0.83	0.86	0.89	1.13	1.23	1.40	a
		3.0	56.0	171.48	216.62	285.03	0.84	0.88	0.91	1.16	1.28	1.37	a
	ZR75-1	1.5	37.0	97.598	163.31	206.35	0.77	0.84	0.87	1.27	1.43	1.65	a
		2.0	49.0	105.69	129.45	160.29	0.77	0.84	0.86	1.20	1.32	1.47	a
		2.5	66.0	98.26	130.57	155.01	0.83	0.86	0.89	1.21	1.31	1.45	a
		3.0	55.0	117.35	155.43	203.08	0.83	0.88	0.89	1.14	1.31	1.42	a

*apparent migration mode – describes the migration mode of cells due to the visual appearance in the images in Figures S6 – S8 and in the supplementary videos 1-9

Video S1: MCF-7_B-collagen.mp4

This video shows MCF-7 cells that had invaded a 1.5 g/l bovine collagen matrix for 72 hours, scale bars are 20 μm.

Video S2: MCF-7_R-collagen.mp4

This video shows MCF-7 cells that had invaded a 1.5 g/l rat collagen matrix for 72 hours, scale bars are 20 μm.

Video S3: MCF-7_RB-collagen.mp4

This video shows MCF-7 cells that had invaded a 1.5 g/l mixed collagen matrix for 72 hours, scale bars are 20 μm.

Video S4: MDA-MB-231_B-collagen.mp4

This video shows MDA-MB-231 cells that had invaded a 1.5 g/l bovine collagen matrix for 72 hours, scale bars are 20 μm.

Video S5: MDA-MB-231_R-collagen.mp4

This video shows MDA-MB-231 cells that had invaded a 1.5 g/l rat collagen matrix for 72 hours, scale bars are 20 μm.

Video S6: MDA-MB-231_RB-collagen.mp4

This video shows MDA-MB-231 cells that had invaded a 1.5 g/l mixed collagen matrix for 72 hours, scale bars are 20 μm.

Video S7: ZR75-1_B-collagen.mp4

This video shows ZR75-1cells that had invaded a 1.5 g/l bovine collagen matrix for 72 hours, scale bars are 20 μm.

Video S8: ZR75-1_R-collagen.mp4

This video shows ZR75-1 cells that had invaded a 1.5 g/l rat collagen matrix for 72 hours, scale bars are 20 μm.

Video S9: ZR75-1_RB-collagen.mp4

This video shows ZR75-1 cells that had invaded a 1.5 g/l mixed collagen matrix for 72 hours, scale bars are 20 μm.
